# Assessing the feasibility of using smartphone data to identify risk of idiopathic pulmonary arterial hypertension

**DOI:** 10.1038/s44325-026-00114-9

**Published:** 2026-03-25

**Authors:** Juan A. Delgado-SanMartin, Merve Keles, Niamh Errington, Narayan Schuetz, Anders Johnson, Varsha Gupta, Steve Hershman, Mark Toshner, Martin R. Wilkins, David G. Kiely, Roger Thompson, Euan Ashley, Dennis Wang, Allan Lawrie

**Affiliations:** 1 National Heart and Lung Institute, Imperial College London, London, UK; 2 Department of Medicine, Stanford University, Stanford, CA USA; 3 Agency for Science, Technology and Research (A*STAR), Singapore, Singapore; 4 University of Texas, Austin, TX USA; 5 Department of Medicine, University of Cambridge, Cambridge, UK; 6 National I/HPAH Cohort Study, Cambridge, UK; 7 NIHR Biomedical Research Centre, Sheffield and University of Sheffield, Sheffield, UK

**Keywords:** Biomarkers, Cardiology, Diseases, Health care, Medical research

## Abstract

Idiopathic pulmonary arterial hypertension (IPAH) is a progressive, life-limiting condition often diagnosed late due to non-specific symptoms and requirement of invasive right heart catheterisation. This pilot study explores the feasibility of using real-world physical activity data from wearable devices and a smartphone app (My Heart Counts) to aid earlier detection. We analysed up to eight years of retrospective data from 109 UK participants, including patients with IPAH, disease controls, and healthy individuals. A classifier trained on pre-diagnostic activity and heart rate, distinguished individuals with IPAH from healthy and disease controls with an ROC AUC of 0.87, improving to 0.94 with in-app questionnaire input. Validation in a matched US cohort yielded an ROC AUC of 0.74. Wearable-derived metrics correlated with clinical 6MWD supporting their potential to complement traditional risk assessment. These pilot findings suggest that digital health tools may support earlier detection and remote monitoring of IPAH warranting larger scale studies.

## Introduction

Idiopathic pulmonary arterial hypertension (IPAH) is a rare, debilitating and progressive life-limiting disease that has no cure and presents a significant healthcare burden^1^. IPAH is diagnosed haemodynamically by invasive right heart catheterisation^1^ at tertiary referral centres. Current treatments, especially when applied early^2^, can extend life and ameliorate morbidity but can be extensive (triple therapy, intravenous treatment, etc.), and have side effects that can affect quality of life (QoL). However, as with most rare diseases, there is often a significant delay (approximately 3 years) from first symptom to diagnosis^3^, and subsequent treatment due to non-specific symptoms^4^. Increased awareness and the growing availability of treatments have led to a parallel increase in diagnostic rates, resulting in a prevalence that has more than doubled over the past 15 years^5^.

We have previously demonstrated that the delay in diagnosis can result in patients presenting with more advanced disease, often complicated by additional co-morbidities, particularly in older patients, and presenting with a high-risk of 1-year mortality^6^. Reducing the time to diagnosis is therefore essential to improve patient outcomes, minimise the number of investigations, and allow treatment initiation at an earlier disease stage, when therapies may be more effective^4^. There are several clinical screening and early detection algorithms in use for patients at risk of PAH, e.g. systemic sclerosis^4^, but screening for patients at risk of idiopathic PAH remains a challenge.

Current care models for IPAH rely on information collected during infrequent clinic visits. Even when visits occur regularly, they are often poorly timed and insufficient for accurate tracking of disease progression^7^. Advances in digital health, including smartphone applications (apps) and wearable technologies, offer the potential to remotely monitor patients and collect longitudinal data in real-world settings^8^. Physical activity is a well-established determinant of cardiovascular health, with higher activity levels associated with improved outcomes and reduced mortality across multiple cardiovascular diseases, while physical inactivity is a major risk factor for the development of heart disease^9^. Despite the growing body of literature on digital health and its impact on care, limited research has explored the utility of using real-world physical activity obtained from smartphones and wearable devices to support early diagnosis of IPAH^10^.

Previous studies in controlled study conditions have reported lower baseline physical activity in patients with IPAH^11,12^, and average step count has been associated with QoL in PAH^12,13^. However, studies using wearables with accelerometers typically have a short period of monitoring, for example, a fixed period of 5 days^11^ or a week^14^, and provide only a snapshot rather than a comprehensive view of patients’ daily lives or their process before and after diagnosis.

The My Heart Counts Cardiovascular Health Study was launched in 2015 as one of the first ‘apps’ to utilise the open source ResearchKit framework from Apple Inc (Cupertino, CA, USA) to facilitate clinical research. The My Heart Counts app (MHC) is free to download and incorporates e-consent, direct photoplethysmogram (PPG) sensor-based measurements of physical activity and fitness, as well as questionnaire assessment of sleep, lifestyle factors, risk perception, mental well-being, overall well-being and a 6-min walk test (6MWT)^15^. MHC has been utilised both in the US^15–18^ and the UK^19^ to conduct real-world, longitudinal studies on cardiovascular and COVID-19-related studies.

We present a pilot study using MHC to leverage passively collected, real-world activity data and questionnaire responses from a cohort of IPAH patients, disease controls, and healthy volunteers with longitudinal data collection spanning 273 days to 8.4 years. Unlike short-term snapshots, this extended longitudinal approach captures natural variations in daily life outside hospital settings, reducing the risk of false indicators that can arise from limited or context-specific measurement. By integrating these long-term data streams with traditional clinical variables, we demonstrated the potential to develop early warning signals, an IPAH classification model, and putative digital biomarkers for continuous, remote patient monitoring.

## Results

### UK My Heart Counts cohort recruitment

**Fig. 1:**
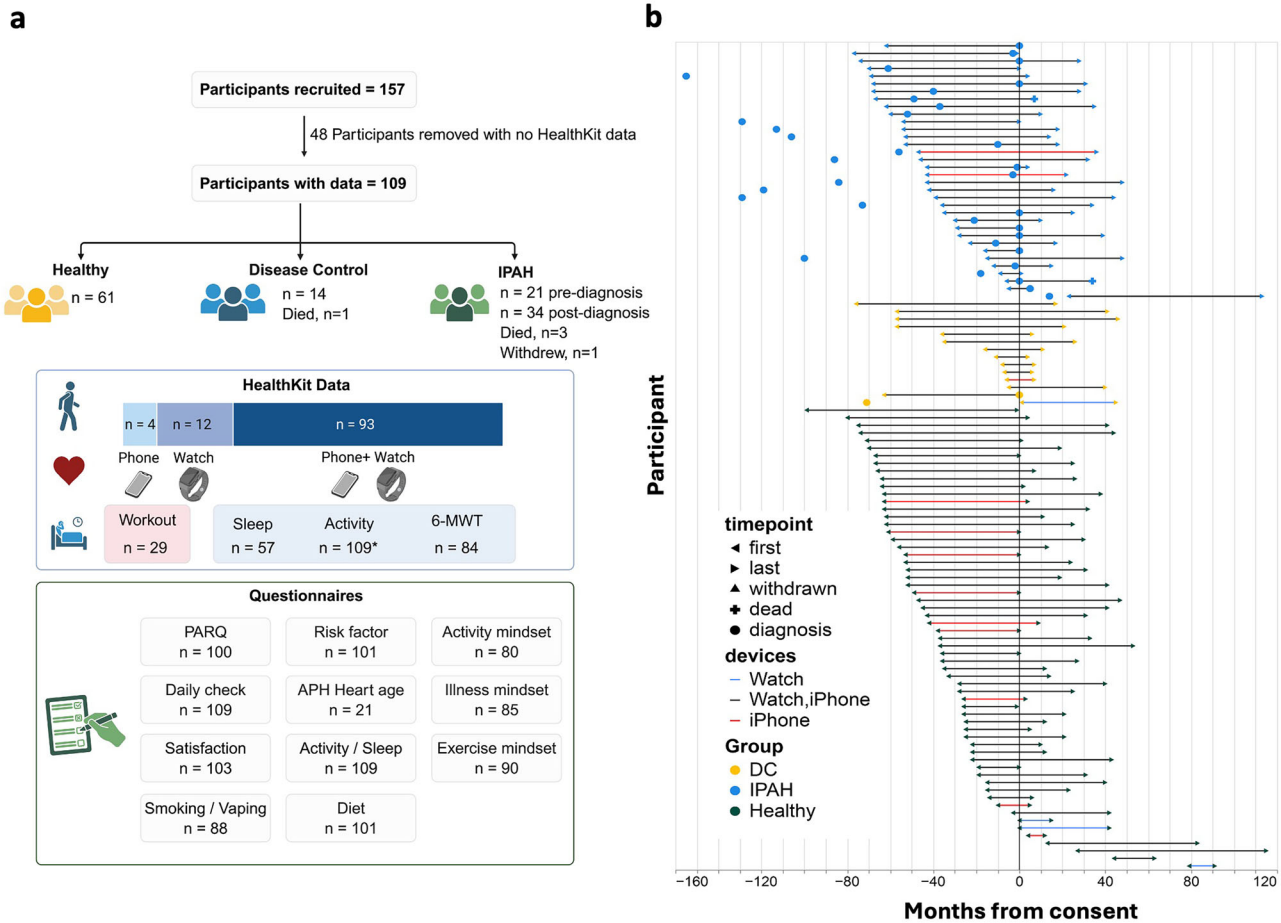
Recruitment and data availability in the UK My Heart Counts cohort. **a** Summary of study participants with available activity data in each group, including the source of data collected (phone and watch). The number of participants contributing HealthKit data and questionnaires is indicated. Data from workouts were not included in analyses (indicated in red box. PARQ Physical Activity Readiness Questionnaire, 6MWT: 6-min walk test. **b** Timeline of activity data relative to consent (0 months) and time of diagnosis. Participants were grouped as idiopathic pulmonary arterial hypertension (IPAH), disease controls (DC), or healthy volunteers. Solid lines indicate data availability within a 6-month sliding window, with line colour denoting the source device.

A total of 157 participants who already owned an iPhone (phone) were enroled in the pilot study. Each participant received instructions to install the My Heart Counts (MHC) app, was provided with an Apple Watch Series 4 (watch), and a study PseudoID to link MHC data to their clinical record. 48 participants were excluded from analysis due to failing to record any HealthKit data. The remaining 109 participants recorded HealthKit data and were included in the analysis. Thirty-four participants were diagnosed with IPAH, with 1 patient withdrawing consent. Fourteen participants were considered as disease controls (DC), comprising 12 post-hospitalisations for severe COVID-19, and 2 patients with suspected IPAH but normal pulmonary artery pressure (<20 mmHg). The remaining 61 participants were considered healthy controls (Healthy). In addition to the HealthKit Activity data, 62 participants performed at least one My Heart Counts 6-min walking test (6MWT), 57 participants provided sleep data, and 29 participants had Apple Watch Workout data (not used) (Fig. 1a). All 109 patients responded to at least 5 questions within the My Heart Counts questionnaires to assess existing levels of physical activity (Physical Activity Readiness Questionnaire, PAR-Q), sleep, diet, health and mindset (exercise, illness, activity) (Fig. 1a). Data were collected between September 2014 and August 2024. From the 33 patients with IPAH, pre-diagnosis activity data were available from 21 individuals and were collected from April 2015 to November 2022. All 33 IPAH patients provided data following diagnosis, with a median of 5.4 years [IQR - 3.4, 6.9] (Fig. 1b). Basic demographics for the whole UK My Heart Counts cohort are shown in Table 1.

**Table 1:** UK My Heart Counts cohort demographics

	IPAH	Healthy	Disease control	Total
**Gender**				
Female	26	41	3	70
Male	8	20	11	39
**Ethnicity**				
White	25	51	10	86
Non-white	3	3	1	7
Unknown	6	7	3	16
**Age at Consent**				
Age (years) Median [min, max]	46 [22, 81]	45 [20, 70]	59 [36, 75]	46 [20, 81]
Unknown	2	14	3	19
**Body Mass Index (BMI)**				
Underweight < 18.5	2	0	0	2
Normal 18.5–24.9	8	8	2	18
Overweight or obese > 25	16	13	2	31
Unknown	8	40	10	58

### UK My Heart Counts IPAH patient clinical demographics

IPAH demographics and clinical phenotype data from diagnostic right heart catheterisation are summarised in Table 2.

**Table 2:** Clinical phenotype data at diagnosis for UK IPAH My Heart Counts participants

**Right heart catheter (RHC)**	
Mean pulmonary artery pressure (mPAP, mmHg)	53.6 ± 15.6
Pulmonary artery wedge pressure (PAWP, mmHg):	8.4 ± 3.5
Pulmonary Vascular resistance (PVR, dynes.sec.cm^−5^)	1042.2 ± 569.2
Cardiac output (CO, l/min)	3.8 ± 1.8
**Lung function**	
Forced expiratory volume in 1 s, percentage of predicted (FEV1% Predicted)	85.8 ± 15.2
Forced vital capacity, percentage of predicted (FVC % Predicted)	93.4 ± 18.3
Transfer factor of the lung for carbon monoxide, percentage of predicted (TCLO% Predicted)	62.6 ± 18.4
**Exercise tests**	
6-min walk test distance (6MWD, m)	450.9 ± 114.9
Incremental shuttle walk test distance (ISWD, m)	514.4 ± 275.1
**BNP/NT-proBNP**	
N-terminal pro-B-type natriuretic peptide (NT-proBNP, pg/ml)	471.2 ± 664.4
B-type natriuretic peptide (BNP ng/L):	195.2 ± 217.4
**WHO Functional class (FC)**	
I – II – III - IV (% of cohort)	6 – 30 – 63 - 1

Values are presented as mean ± standard deviation (std).

### Phone-derived HealthKit activity metrics identifies differences between participant groups

**Fig. 2:**
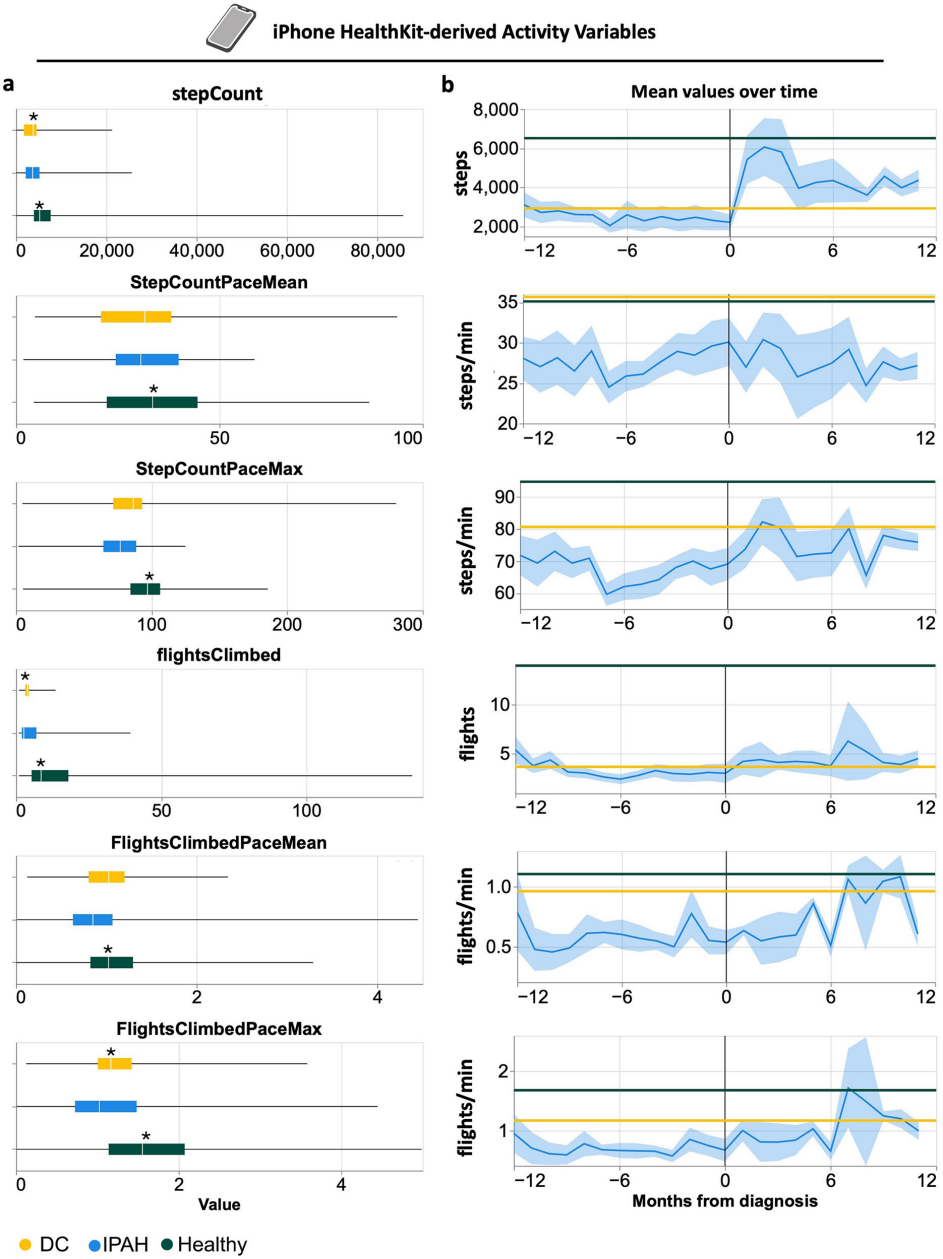
Longitudinal trends of phone HealthKit-derived physical activity variables from IPAH patients. **a** Box plots showing the distribution of HealthKit-derived physical activity metrics in IPAH cases prior to diagnosis. Metrics include total step count (stepcount), mean and maximum step pace (StepCountPaceMean and StepCountPaceMax, steps/min), total flights climbed (flightsClimbed), and mean and maximum pace of flights climbed (flightsClimbedPaceMean and flightsClimbedPaceMax, flights/min). **b** Longitudinal trends in monthly mean values of the same activity metrics from 12 months before to 12 months after diagnosis (month 0), with shaded areas representing 95% confidence intervals. Lines for disease controls (DC) and healthy volunteers (Healthy) represent group averages across the entire 24-month period. Statistical comparisons between IPAH cases and controls were performed using the Mann–Whitney U test; *p* < 0.05 was considered significant.

To determine the utility of activity data captured by the phone’s accelerometer, we first isolated data captured by each device (iPhone and Apple Watch). The median value and interquartile range of each phone-measured variable, and comparisons between healthy or disease controls (DC), with IPAH patients across all time points are provided in Table 3. There was a significant reduction in the number of steps (*stepCount*), the calculated average walking pace (StepCountPaceMean, Eq. (4)), and the maximum walking pace (StepCountPaceMax, Eq. (5)) with IPAH compared to healthy controls. Similarly, for the number of flights of stairs climbed (*flightsClimbed*), mean pace at climbing stairs (FlightsClimbedPaceMean, Eq. (7)) and max pace at climbing stairs (FlightsClimbedPaceMax, Eq. (8)), patients with IPAH were significantly lower than healthy controls. When compared to diseased controls, there was a significant reduction in the number of steps (*stepCount*), number of flights climbed (*flightsClimbed*), and the max pace at climbing stairs (FlightsClimbedPaceMax) (Fig. 2a).

**Table 3:** Phone activity metrics

Variable				*p*-values (adj)		*n*
median [IQR] /mean ± SD	DC	Healthy	IPAH	IPAH v DC	IPAH v Healthy	IPAH: Healthy:DC
*FlightsClimbed*	5.27 ± 6.90	10.27 ± 17.97	6.19 ± 11.87	<0.0001	<0.0001	29:54:11
FlightsClimbedPaceMax	1.2 [0.61, 1.88]	1.35 [0.63, 2.5]	0.83 [0.29, 1.62]	0.0307	0.00194	29:52:11
FlightsClimbedPaceMean	0.88 [0.49, 1.4]	0.94 [0.48, 1.58]	0.63 [0.25, 1.13]	0.477	0.0144	29:52:11
*StepCount*	3323.0 [1657.0, 5490.0]	4620.0 [2285.0, 7650.0]	2596.0 [982.0, 5358.0]	<0.0001	<0.0001	32:57:13
StepCountPaceMax	81.94 [54.7, 103.1]	101.2 [73.64, 115.25]	86.92 [56.5, 104.37]	0.464	0.0108	33:57:13
StepCountPaceMean	27.05 [16.16, 40.25]	32.26 [20.72, 45.89]	30.02 [19.37, 42.34]	0.563	<0.0001	33:57:13

Continuous data are presented as median [interquartile range, IQR] and categorical data are presented as mean ± standard deviation (SD). Statistical significance was assessed using the Mann–Whitney U test (p-adjusted, *p*-adj). The values are adjusted for ethnicity, age, sex and BMI. HealthKit variables are in italics, calculated variables are plain text.

Within the patients with IPAH, we next examined whether activity patterns showed differences pre- and post-diagnosis. After diagnosis, there was a significant increase in *stepCount* and *flightsClimbed*, in addition to their relative mean and max paces (Fig. 2b). There was also a significant reduction in both resting and walking heart rates (Fig. 2b, Table S1).

### Watch-derived HealthKit activity, heart rate and sleep metrics reveal differences between participant groups

Participants were offered an Apple Watch at the point of recruitment. Since only a few participants already owned a device, pre-diagnostic smartwatch-derived (PPG-based) HealthKit features were sparser compared to phone-derived data. However, to determine whether the watch-collected data were comparable to the phone data, we first examined the overlapping HealthKit variables. Consistent with the phone-derived data, we observed a marked difference between IPAH and healthy controls in StepCountPaceMean (Eq. (4)), *flightsClimbed*, and FlightsClimbedPaceMean (Eq. (7)) (Table 4, Fig. S1).

**Table 4:** Watch activity and sleep metrics

variable				*p*-adjusted		*n*
median [IQR] /mean ± SD	DC	Healthy	IPAH	IPAH v DC	IPAH v Healthy	IPAH: Healthy:DC
*ActiveEnergyBurned*	436.8 [318.77, 595.34]	501.95 [317.44, 694.99]	353.7 [234.41, 491.13]	<0.0001	0.473	30:50:11
*AppleStandTime*	1.63 [1.1, 2.22]	1.82 [1.1, 2.58]	1.2 [0.65, 1.9]	0.334	0.192	28:47:10
*Asleep*	7.37 [6.3, 8.19]	6.35 [4.15, 8.07]	6.63 [4.97, 7.83]	0.0104	0.549	09:22:03
*Awake*	0.59 [0.38, 0.91]	0.15 [0.08, 0.29]	0.41 [0.18, 1.08]	NA	NA	06:04:01
*BasalEnergyBurned*	1829.83 [1592.55, 2124.12]	1642.18 [1463.31, 1809.07]	1680.32 [1472.62, 1863.12]	<0.0001	<0.0001	30:50:10
CardiacEffort	0.07 [0.05, 0.13]	0.06 [0.04, 0.17]	0.07 [0.05, 0.18]	<0.0001	0.0024	27:44:09
*FlightsClimbed*	18.8 ± 38.2	16.5 ± 20.9	10.3 ± 22.2	0.0188	0.332	28:47:09
FlightsClimbedPaceMax	2.35 [0.87, 5.08]	3.08 [1.3, 4.39]	1.36 [0.54, 3.33]	0.646	0.0066	28:46:09
FlightsClimbedPaceMean	1.72 [0.78, 2.97]	1.84 [0.99, 2.88]	1.07 [0.48, 2.1]	0.0116	0.0182	28:46:09
*HeartRate*	79.64 [70.72, 88.93]	83.53 [72.88, 99.44]	78.53 [70.38, 87.99]	0.271	0.0012	31:49:11
HeartRateReserve	12.06 [7.92, 17.97]	19.06 [10.72, 34.63]	12.48 [8.51, 18.66]	0.0902	<0.0001	31:47:10
*HeartRateVariabilitySDNN*	24.72 [18.42, 32.84]	37.95 [30.07, 49.34]	29.93 [21.44, 44.27]	0.356	0.0006	29:47:11
*RestingHeartRate*	66.0 [59.0, 74.0]	62.0 [57.0, 68.0]	64.0 [57.0, 72.0]	<0.0001	0.828	31:47:10
*StepCount*	4693.0 [3075.0, 7196.0]	7185.0 [4313.0, 10780.0]	4861.0 [2613.0, 7879.0]	<0.0001	0.0384	29:47:10
StepCountPaceMax	97.89 [72.63, 110.85]	118.18 [101.14, 130.0]	102.86 [81.64, 115.16]	<0.0001	0.0183	29:47:10
StepCountPaceMean	34.24 [22.21, 46.0]	46.25 [34.76, 58.5]	38.05 [27.97, 46.88]	<0.0001	0.856	29:47:10
*VO*_*2*_*Max*	23.8 [19.08, 45.82]	37.4 [31.78, 40.33]	26.26 [22.3, 31.95]	<0.0001	<0.0001	22:39:10
WalkingHeartRateAverage	97.0 [84.5, 108.0]	96.5 [86.5, 107.0]	99.0 [88.0, 110.0]	0.0008	0.062	30:46:11

Continuous data are presented as median [interquartile range, IQR] and categorical data are presented as mean ± standard deviation (SD). Statistical significance was assessed using the Mann–Whitney U test (*p*-adjusted). The values are adjusted for ethnicity, age, sex and BMI. HealthKit variables are in italics, calculated variables are plain text. No statistical test was applied where sample sizes were insufficient.

**Fig. 3:**
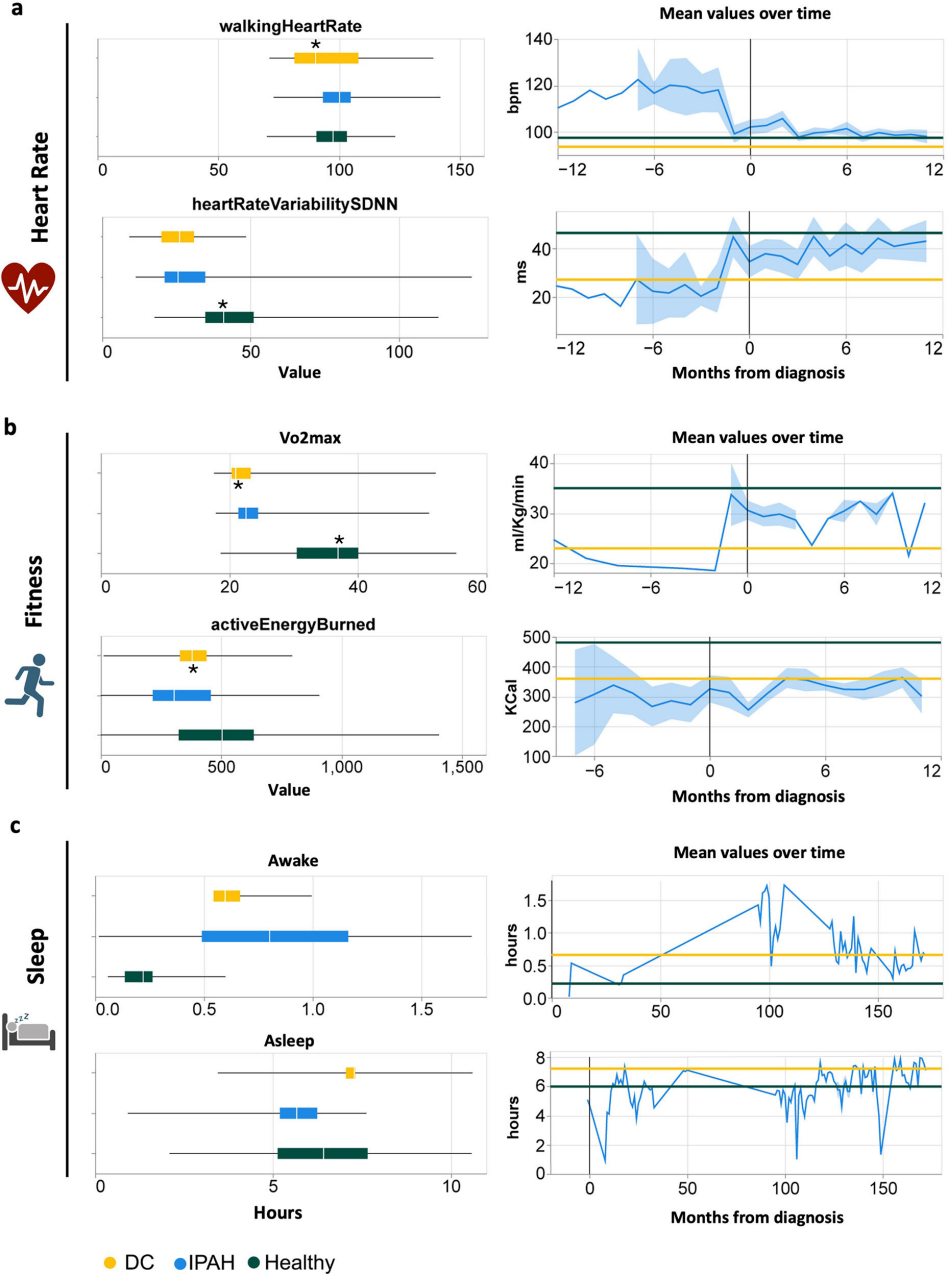
Longitudinal trends of cardiovascular, fitness, and sleep metrics derived from watch in IPAH patients. **a** Heart rate metrics: Box plots (left) show distributions of walking heart rate (*walkingHeartRate*), heart rate variability (*heartRateVariabilitySDNN*) in IPAH cases prior to diagnosis. Line plots (right) display monthly mean values from 12 months before to 12 months after diagnosis, with shaded areas representing 95% confidence intervals. **b** Fitness metrics: Box plots (left) show distributions of estimated VO₂ max (*VO₂Max*) and active energy burned (*activeEnergyBurned*, KCal). Line plots (right) show longitudinal trends in monthly mean values across the same 24-month window. **c** Sleep metrics: Box plots and line plots illustrate distributions and temporal patterns of *Awake* and *Asleep* hours, analysed both in 5-h intervals and monthly averages. Groups include IPAH patients (blue), disease controls (DC, yellow), and healthy volunteers (green). Statistical comparisons were performed using the Mann–Whitney U test; *p* < 0.05 was considered significant.

Examination of the data obtained from the PPG, e.g. heart rate data, identified significant differences between healthy controls and patients with IPAH in average heart rate (*HeartRate*), walking heart rate (*walkingHeartRate*, Fig. 3a), heart rate variability (*HeartRateVariabilitySDNN*, Fig. 3a) and HeartRateReserve ((Eq. (1)), Fig. S2). Similarly, there were significant differences between disease controls and patients with IPAH in resting heart rate (*restingHeartRate*) and heart rate reserve (HeartRateReserve) (Fig. S2).

Watch-derived estimates of active energy burned (calories) and VO_2_max can provide an indication of fitness. There were significant differences in these metrics when comparing both the healthy and disease controls with patients with IPAH (Fig. 3b). There were similar significant differences in Cardiac Effort (*averageHeartRate*/time spent active, Table 4) and basal energy burn (*basalEnergyBurned*).

Data on sleep metrics were relatively sparse compared to other PPG-heart rate-derived data (in the UK, there were 11 participants with 210 ± 178 days, in the US, just 7 participants with 162 ± 156 days’ worth of data). However, analysis of sleep data identified a significant difference in the time patients with IPAH spent *Awake* (56 min) at night compared to the control groups (15 min) (Fig. 3c).

### Differences in daily, weekly and seasonal patterns of physical activity and heart rate

**Fig. 4:**
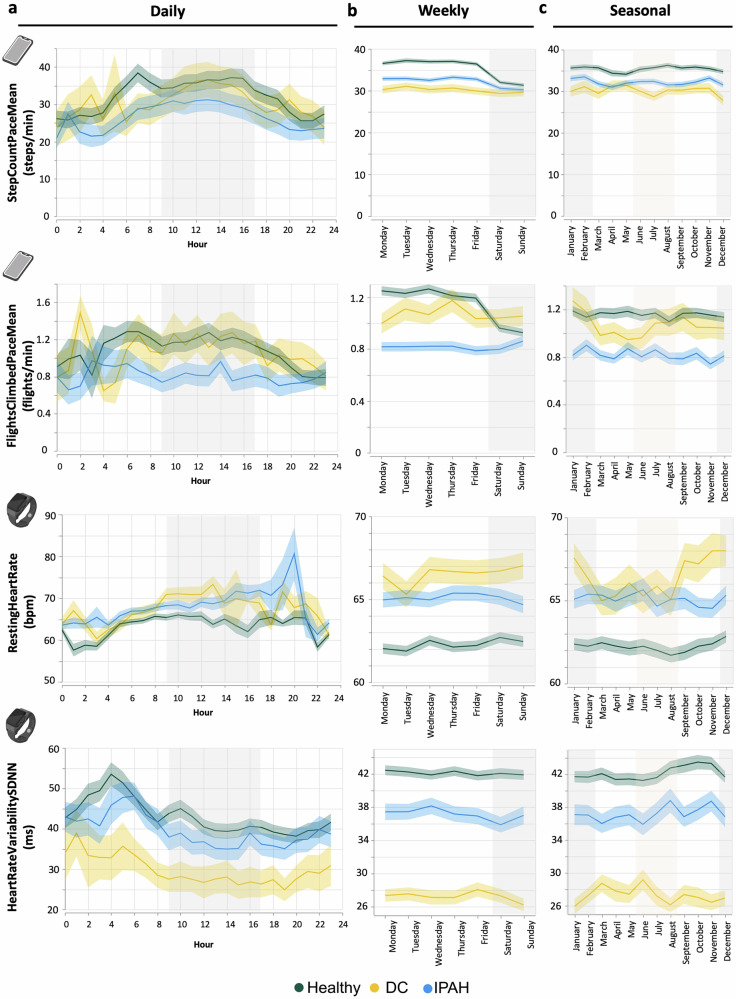
Patterns of activity and cardiovascular physiology across daily, weekly and seasonal timescales. **a** Daily patterns shown across 24 h, with a shaded area (grey) for working days. **b** Weekly patterns across days of the week, with shading highlighting workdays (white) and weekends (grey). **c** Seasonal patterns across months, with shading indicating winter (grey), summer (orange), and spring/autumn (white). Groups include IPAH patients (blue), disease controls (DC, yellow), and healthy volunteers (green).

To maximise the utility of the long-term data collection, we next explored differences in daily (Fig. 4a), weekly (Fig. 4b) and seasonal (Fig. 4c) patterns between IPAH patients and the healthy and disease control groups. Compared to both disease controls (DC) and healthy individuals, IPAH participants exhibited significantly lower average step (StepCountPaceMean, Eq. (4)) and flights climbed (FlightsClimbedPaceMean, Eq. (7)) pace during the day (Error! Reference source not found.a). Patients with IPAH also recorded significantly higher resting heart rate (*RestingHeartRate*) and lower heart rate variability (*HeartRateVariabilitySDNN*) compared to healthy controls (Fig. 4a).

Weekly patterns of activity highlight that healthy controls demonstrated a reduced step count pace (StepCountPaceMean) and flights climbed pace (FlightsClimbedPaceMean) at the weekends, compared to weekdays (Fig. 4b, Table S2). There was a less notable difference between weekday and weekend activity in patients with IPAH. However, these metrics were significantly reduced compared to healthy controls (Fig. 4b). During the weekdays, patients with IPAH demonstrated a more consistent impairment in both step count pace (StepCountPaceMean) and flights climbed pace (FlightsClimbedPaceMean). There was less variability in both resting heart rate (*restingHeartRate*) and heart rate variability (*HeartRateVariabilitySDNN*) across the week for all participant groups (Error! Reference source not found.b).

Seasonal analyses further emphasised the differences between patients with IPAH and either disease controls or healthy individuals (Fig. 4c), underscoring the functional impairment of patients with PAH (Table S2).

### Contrasting responses to lifestyle, activity and mindset questionnaires

In addition to analysing the activity data, heart rate, and sleep data, over the first 7-days following enrolment into the MHC study, participants were requested to complete a number of surveys (Tables S3–4) including Physical Readiness Questionnaire (PAR-Q), Activity and Sleep survey^20^, Cardio Diet Survey, Well-being and Risk perception Survey^21^, as previously described^15,18^ (Tables S4–8). In addition, participants were also asked to complete three mindset questionnaires, the Adequacy of Activity Mindset Measure (AAMM)^22^, illness mindset^23^ and exercise^24^ process mindset to assess mindsets about maintaining good. Statistical analysis across all questionnaire responses comparing patients with IPAH, healthy controls and DC responders identified 15 questions (excluding questions directly related to cardiovascular disease and prescription drugs) with significant differences (Table S9). The most prominent differences were related to risk factors and whether participants were physically capable of working.

**Fig. 5:**
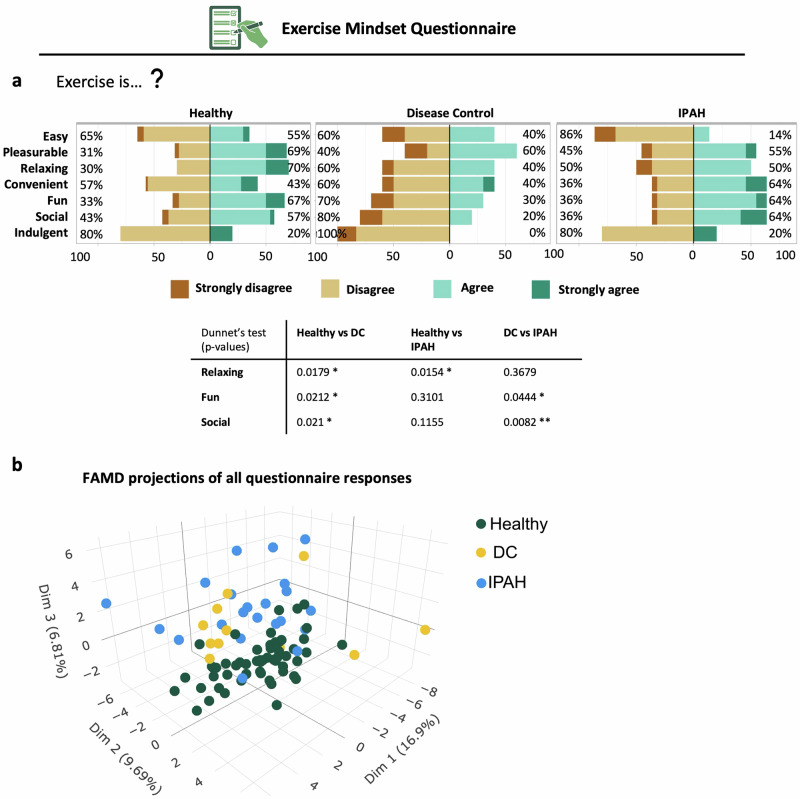
Perceptions of exercise and questionnaire response differences across healthy, DC and IPAH cohorts. **a** Responses to the *Exercise Mindset Questionnaire* evaluating perceptions of exercise as easy, pleasurable, relaxing, convenient, fun, social, and indulgent. Bars represent the proportion of participants within each response category (strongly disagree, disagree, agree and strongly agree). Group comparisons using Dunnett’s test identified significant differences in perceptions of exercise as relaxing, fun and social. **b** Factor Analysis of Mixed Data (FAMD) projections of all My Heart Counts questionnaire responses, showing clustering of individuals by cohort (Healthy = green, DC = yellow, IPAH = blue).

Within the mindset questionnaires, there were group-level differences in beliefs about physical activity and its health implications (Fig. S3a). Healthy participants were more likely to perceive their current activity levels as beneficial for body weight and disease prevention. In contrast, patients with IPAH expressed uncertainty or disagreement regarding the health benefits of their activity levels, with a notable proportion indicating that physical activity had minimal impact on disease risk or muscle strength. The disease control group showed intermediate responses, aligning more closely with healthy participants on some items but diverging on others, particularly regarding perceived risk reduction (Fig. S3a). Responses to the illness mindset questionnaire highlighted that healthy and disease control participants were more likely to agree that lifestyle changes can prevent illness and that stress contributes to heart disease. IPAH participants, however, showed lower agreement with these statements, and a higher proportion attributed illness to genetic factors (Fig. S3b). Responses to the Exercise Mindset Questionnaire demonstrated significant differences in perceptions of exercise across three groups (Fig. 5a). Healthy participants were most likely to strongly agree that exercise is easy (65%) and pleasurable (72%), compared to DC (55% and 60%, respectively) and IPAH (14% and 28%, respectively). IPAH participants consistently reported lower agreement across all positive exercise attributes, particularly for ‘easy’ and ‘fun’. There were significant differences across the groups in perceptions of exercise as relaxing, fun, and social. IPAH participants rated exercise as significantly less relaxing and less fun compared to both healthy and DC groups (Fig. 5a).

To determine whether the distribution of questionnaire responses aligned with the participant groups, we performed a dimensional reduction of the data using a Factor Analysis of Mixed Data (FAMD). We found a significant separation in the distribution of responses that aligned with their participant group. 3D projection revealed distinct clustering of responses, with IPAH participants forming a separate cluster from Healthy and DC groups along Dim1 (16.5%), Dim2 (9.6%) and Dim3 (6.5%), suggesting divergent mindset and profiles of patients with IPAH towards lifestyle factors, chronic disease, and mindset (Fig. 5b).

### Remote physical activity, heart rate and questionnaire metrics correlate with clinical risk variables

Clinical risk scores are used to predict the 1-year survival for patients with IPAH (compared in Yogeswaran et al.^25^), with specific algorithms favoured by different regions or countries. However, most include common variables such as 6-min walk distance, NT-proBNP/BNP concentration and WHO functional class with some allowance for missing data. These clinical risk scores are beneficial when assessing patients’ disease severity at diagnosis and treatment response, but are limited by the ‘snapshot’ data collection. To address this, we examined the correlation between the watch and phone activity metrics collected within MHC and clinical scores collected in the hospital (ERS/ESC 4-strata risk score^1^).

**Fig. 6:**
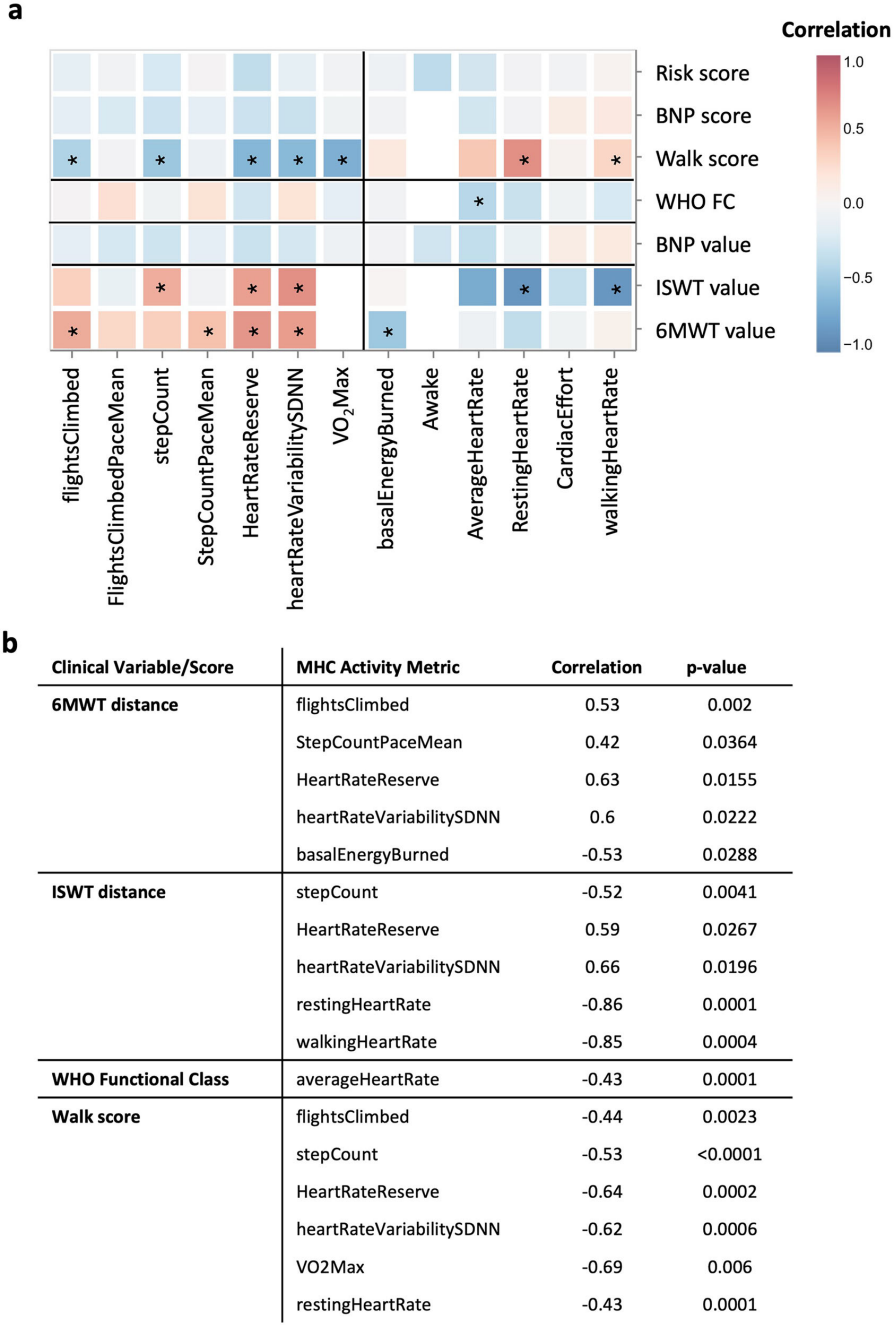
Correlation between clinical parameters and pre-diagnosis activity metrics. **a** Heatmap showing Pearson correlation coefficients between digital health metrics and clinical parameters from the My Heart Counts dataset. Colour intensity reflects the strength and direction of correlation (range: −1 to +1), with red indicating positive and blue indicating negative associations. *p* < 0.05 was considered significant, and correlations $\rho > 0.4.$ **b** Correlation coefficients and *p*-values for 6-min Walk Test (6MWT), Incremental Shuttle Walk Test (ISWT), and WHO Functional Class and ERS calculated walk scores (Walk score).

In clinical exercise test data were available from two walk tests: the Incremental Shuttle Walk Test (ISWT) and the 6-min Walk Test (6MWT). Although the MHC app also records a remote 6MWT, poor concordance with clinical tests (primarily conducted during the COVID-19 pandemic), and limited test repetitions by individual made direct correlation unfeasible in this study. We therefore generated a correlation matrix to examine the relationship between clinical variables and MHC data recorded from patients with IPAH (Fig. 6a, b). The clinical 6MWT distance correlated significantly with *flightsClimbed*, StepCountPaceMean (Eq. (4)), HeartRateReserve (Eq. (1)), and heart rate variability (*heartRateVariabilitySDNN*), and *basalEnergyBurned* (Fig. 6). The ISWT distance similarly had a significant correlation with *stepCount*, HeartRateReserve, heart rate variability (*heartRateVariabilitySDNN*) *restingHeartRate* and *walkingHeartRate* (Fig. 6). Additionally, MHC recorded *averageHeartRate* correlated (Fig. 6) and the chronic illness survey (Table S10) correlated with WHO functional class. We next categorised the 6MWT distances and NT-proBNP/BNP levels based on thresholds from the ERS/ESC guidelines^1^ and the ISWT distance using Lewis et al^26^. Significant correlations were observed between the risk walk scores and *flightsClimbed*, *stepCount*, HeartRateReserve, heart rate variability (*heartRateVariabilitySDNN*), *VO*_*2*_*Max*, and *restingHeartRate* (Fig. 6). While individual activity and heart rate metrics showed significant correlations with variables of the ERS/ESC 4-strata risk score, no significant correlation was observed between the combined risk score and any single activity or heart rate variable. However, a significant correlation was found between the combined risk score and responses from the chronic illness and exercise mindset questionnaires metric, with the exception of three single questions (Table S10)

### Classifying patients with IPAH using smartphone acquired data

**Fig. 7:**
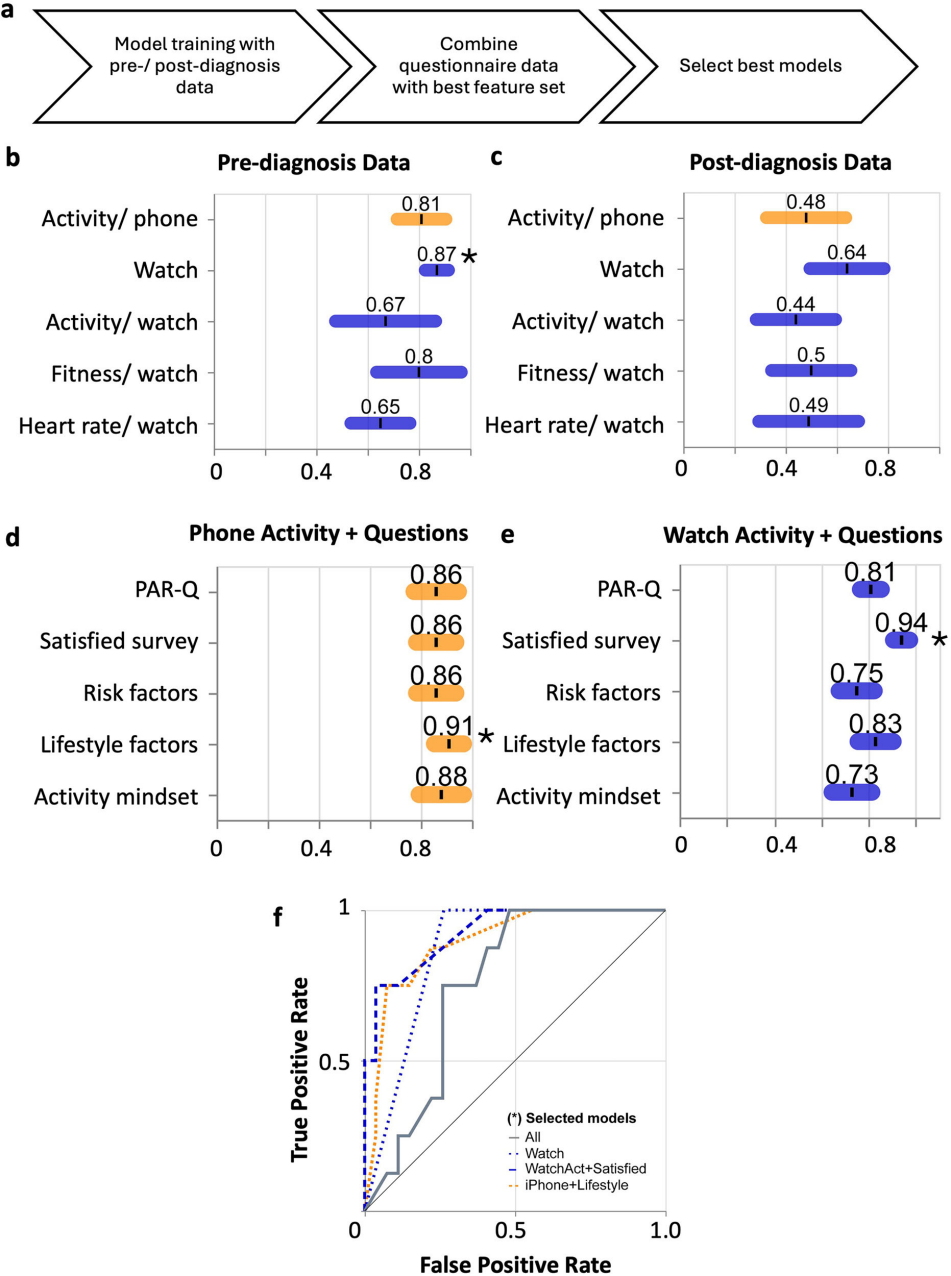
Internal validation (UK Cohort) of XGBoost models using digital activity and questionnaire data. **a** Schematic overview of the model training pipeline, including integration of pre- and post-diagnosis activity data, questionnaire responses, and feature selection to identify optimal models. **b**, **c** Receiver operating characteristic (ROC) area under the curve (AUC) values (mean ± standard deviation) for models trained on all activity data (pre- and post-diagnosis), showing the highest performance for all watch-derived metrics pre-diagnosis (AUC = 0.87 ± 0.07, *n* = 8:46/5:19, IPAH:Rest for train/test). ROC AUCs for models trained on pre-diagnosis activity data only, with watch-based features, again yielding the best performance. **d** ROC AUCs for models combining pre-diagnosis phone activity data with individual questionnaire domains, including PAR-Q, satisfaction survey, risk factors, lifestyle factors, and activity mindset. The best-performing combination was with lifestyle factors (AUC = 0.91 ± 0.09, *n* = 23:57/11:24). **e** ROC AUCs for models combining pre-diagnosis watch activity data with questionnaire domains. The highest performance was achieved when combined with the satisfaction survey (AUC = 0.94 ± 0.07, *n* = 24:56/10:25). **f** ROC curves for selected models (marked with * in **d**, **e**), illustrating classification performance.

Finally, we trained a binary classification model to classify patients with IPAH from the combined disease and healthy controls using data collected solely from the MHC app. We carried out two sets of validations: an internal validation based on the UK data only and an external validation using unseen USA data (see complete modelling strategy in Fig. S6). We trained two models using either XGBoost or a linear support vector machine (SVM) (Fig. 7a). We separated the analysis into pre- and post-diagnosis to isolate diagnosis from any treatment effect to determine whether there was any potential for an early detection tool. Using pre-diagnosis data alone, we achieve a Receiver Operating Characteristic Area Under the Curve (ROC AUC) 0.81 *±* 0.12 using phone data, with the best performance of 0.87 *±* 0.08 obtained using all watch metrics (Fig. 7b). Using post-diagnosis data, we only achieved a ROC AUC of 0.48 *±* 0.18 or phone data, with the best performance of 0.64 *±* 0.17 again achieved using watch data (Fig. 7c, Table S11).

Focusing on the pre-diagnostic data, we next tested whether the addition of the questionnaire responses highlighted in Fig. 5 to the activity metrics for phone (Fig. 7d) and watch (Fig. 7e) would add to the performance of the pre-diagnosis classifier. We focused on the inclusion of five questionnaires: PAR-Q (physical activity readiness survey^27^), Satisfied survey, Risk factors (risk factors associated with cardiovascular disease), Lifestyle factors (e.g. diet and sleep), and the combined mindset (mindset towards physical activity), complete details in Tables S4–8). The addition of either questionnaire responses added to the performance of the phone pre-diagnosis activity data enhanced performance (Fig. 7d, f), with lifestyle factor responses providing a significant improvement to a ROC AUC of 0.91. The addition of questionnaire responses to watch activity metrics provided a boost in performance over watch data alone, with the life satisfaction survey responses providing a significant increase in performance, providing an ROC AUC of 0.94 (Fig. 7e, f). The most important features are related to walking or climbing stairs, highlighting the value of phone metrics alone (Table 12).

### Testing and refining the IPAH classifier using an external US My Heart Counts cohort

We next tested the performance of the IPAH classifier trained on the UK IPAH MHC cohort on an extract of PAH patients obtained from the US MHC cohort. During initial quality control assessment of the US data, we noted significant drift in the data, most notably in the levels of physical activity performed within the two cohorts (Fig. S7). While the directionality of the differences observed in activity metrics between the UK and US IPAH patients was conserved, the magnitude of changes was significantly different. For example, for the variable StepCountPaceMax (Eq. (5)), the median and IQRs were 112 [100, 123] steps/minute for UK participants compared to 86 [56, 112] steps/minute for US participants (Fig. S8 for phone and Fig. S9 for watch).

**Fig. 8:**
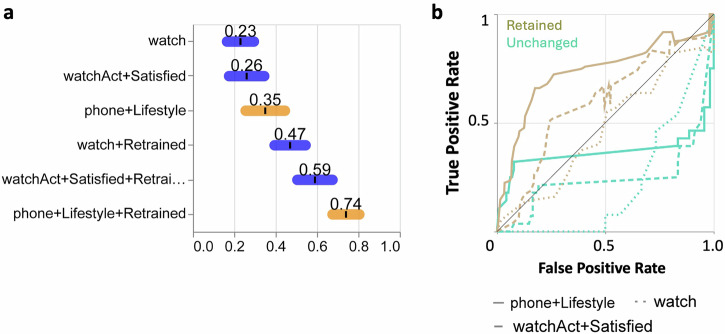
External validation (US Cohort) of XGBoost models using digital activity and questionnaire data. **a** Receiver operating characteristic (ROC) area under the curve (AUC) values (mean ± standard deviation) for models trained on all activity data, showing the highest performance for watch or phone-derived metrics combined with questionnaire data. The highest performance was achieved when combining the phone with the lifestyle surveys (AUC = 0.74 ± 0.02, *n* = 34:81/ 28:115, IPAH:Rest for train/test). **b** ROC curves for selected features (phone+lifestyle, watch and watchAct+Satisfied) illustrating classification performance.

As shown in Fig. 8a, prior to retraining, the model performed with an ROC AUCs < 0.5. To overcome this regional data drift and to provide a more generalised model, we incorporated 20% of the US data into the UK dataset. The retrained XGBoost model (UK + 20% US data) using five-fold cross-validation (Fig. S10) when applied to the remaining US data (all models - watch, phone activity +/- questionnaires) showed improved performance, reaching a ROC AUC of 0.74 ± 0.02 for phone + Lifestyle survey (Fig. 8a, b). No further improvement was observed when including more than 20% of the US data with the UK data for model training (Fig. S11).

## Discussion

Our study highlights the potential of leveraging real-world activity and questionnaire data from smartphones and wearable devices to improve early detection and longitudinal monitoring of PAH. Our longitudinal data (up to 8 years) obtained via the My Heart Counts iOS app demonstrates the utility of using digital biomarkers to distinguish patients with IPAH from healthy and disease control groups, as well as tracking disease progression pre- and post-diagnosis.

We opted for a simplified framework, employing a basic feature extraction method (statistical descriptors and Fourier transform) alongside a relatively straightforward machine learning (ML) approach (XGBoost). We chose this approach to mitigate the challenges posed by the inherent noise and variability encountered in real-world data. Such that we aimed to establish a robust baseline and gain a deeper understanding of the underlying data patterns before progressing to more sophisticated longitudinal clustering approaches (Latent Class Analysis or Growth Mixture Modelling), or more complex feature extractions like wavelet-based features, which would require larger datasets. Our findings indicate that simple activity metrics derived from a smartphone, such as step count, walking pace (gait speed), and pace at climbing flights, can provide valuable insights into physical activity patterns associated with IPAH. Unsurprisingly, reduced activity levels and a slower pace were evident in patients with IPAH pre-diagnosis compared to healthy individuals. These observations align with the known functional limitations caused by the disease and underscore the potential of passive digital phenotyping in aiding with early detection and monitoring of IPAH. However, further work is required in larger datasets to identify IPAH-specific patterns of change from more common cardiovascular diseases, and/or non-cardiovascular disease causes (e.g. musculoskeletal) of changes in activity and fitness.

Importantly, pre-diagnosis metrics, and the addition of wearable data showed promise for predicting IPAH diagnosis, achieving an area under the curve (AUC) of 0.87 ± 0.07 for the watch-only model, and reaching 0.94 + 0.07 when adding Activity Satisfaction surveys. While watch-based metrics resulted in better classification results, phone-derived metrics performed comparably (AUC 0.91 ± 0.09) and offer some advantages, such as a higher adoption rate. Even though smartphones lack direct physical measures such as heart rate, their near-universal and long-term use enables retrospective data collection, providing an opportunity to understand disease-related patterns years before diagnosis. Moreover, smartphones represent a more accessible and balanced solution across socio-economic backgrounds, making them a practical tool for population-level digital phenotyping. However, the addition of heart rate measures, including newer blood pressure alerts, may offer a higher degree of specificity to cardiovascular classifiers.

Our study highlighted significant differences in physical activity across different populations (UK vs US cohorts), including a different distribution of physical activity levels and cardiovascular comorbidities that are important to address. These differences resulted in poor performance of the initial UK model in the US population. To overcome this challenge, we retrained a model incorporating 20% of the US cohort into the UK cohort, to generate a more generalisable model that achieved a ROC AUC of 0.74 ± 0.11 for identifying IPAH. These findings suggest that smartphone and wearable devices could play a role in identifying at-risk individuals and support improving diagnostic delays for IPAH.

Post-diagnosis, patients with IPAH demonstrated significant improvements in activity metrics, likely reflecting the benefit of treatment, consistent with other similar studies^28,29^. Key indicators such as walking pace and heart rate reserve approached levels observed in healthy controls. Integrating activity and heart rate metrics with patient-reported data further enhanced model performance, particularly for lifestyle and mindset-related surveys. These results underscore the value of incorporating patient perspectives to contextualise activity data, offering a more comprehensive understanding of their health status. Furthermore, psychological and lifestyle factors, including perceptions of physical capability and attitudes toward chronic illness, may provide additional diagnostic and prognostic insights.

While our study demonstrates the feasibility of using wearable and smartphone data for IPAH monitoring, several limitations should be acknowledged. Firstly, pre-diagnostic data capture was limited for certain metrics, particularly those related to overnight sleep measurements. Irregular gaps in data may reflect disease-related factors, individual habits, or random patterns. Our study design mitigates the impact of such gaps, if there was some device usage in the months immediately before and after diagnosis. However, extended gaps, lasting over a month or spanning multiple consecutive months, can complicate the interpretation of the results and limit longitudinal insights. Addressing these challenges requires more granular, patient-level analysis, which in turn requires a larger and more diverse participant dataset. Moreover, there are important challenges in comparing activity data collected from the UK and US populations, particularly when we consider expanding to other form of pulmonary vascular disease, and common cardiovascular disease co-morbidities. Differences in lifestyle, including but not limited to travel habits, may contribute to variability between cohorts. The composition of control groups also differed with the US cohort including more non-PAH cardiovascular patients, highlighting the need for larger, more generalised cohorts and analyses. Furthermore, IPAH cases within the US population were self-reported, whereas all UK cases were confirmed at expert PH centres, introducing another potential for inaccuracies. Despite these marked differences, including a transfer of just 20% of the US data to the UK cohort, produced a more reproducible model emphasising the opportunity that exists in harnessing these data. Finally, as highlighted above, the observed decline in activity and heart rate signals is likely not all specific to IPAH and could resemble those seen in other cardiovascular conditions, such as atrial fibrillation or cardiac arrest^30,31^. Identifying unique patterns that can help introduce more disease-specific patterns will require larger, more general datasets and may require the inclusion of other data obtained from wearable devices, e.g. ECG.

This study was designed as proof-of-concept to evaluate whether data collected via a smartphone app could support the identification of individuals at increased risk of IPAH. While our findings highlight distinct patterns in IPAH compared to healthy controls that may support this goal, similar impairments may occur in other cardiovascular conditions. Future work should focus identifying which biomarkers/biomarker patterns are unique, and which are more general. This will also by aided by integrating these with PPG-derived ECG signals and coding data from electronic healthcare records, including what clinical tests have been done, and when they were performed. Integrating these coding data, and in future, the result data from e.g., BNP, transthoracic echocardiography that are often performed many months prior to diagnosis could improve both sensitivity and specificity and support multi-disease classifiers. These non-invasive measures hold promise as a scalable, patient-centred solution for empowering patients and doctors to track disease progression more closely. However, further research and validation in larger, more diverse and linkable cohorts are required before we can fully harness their potential. These studies are critical if data from smartphones and wearables are to be incorporated into routine clinical care.

## Methods

### UK participant recruitment

157 participants were recruited from either the Sheffield Teaching Hospitals Observational Study of Pulmonary Hypertension, Cardiovascular and other Respiratory Disease (STH-ObS) (UK REC Ref 18/YH/0441), or the UK National Cohort of idiopathic and heritable IPAH study (H/IPAH Cohort) (NCT019072950) following informed written consent across four specialist IPAH centres in the UK (Royal Hallamshire Hospital (Sheffield), Hammersmith Hospital (London), Royal Papworth Hospital (Cambridge), and Royal United Hospital (Bath)). Eligible participants (over the age of 18 years) in possession of an Apple iPhone 6 or later were provided with a study pseudo-ID, offered an Apple Watch Series 4, invited to download the My Heart Counts iOS app from the Apple App Store (https://apps.apple.com/us/app/myheart-counts/id972189947), and provide additional consent into the My Heart Counts study (NCT03090321). Baseline demographics, medical history, and relevant clinical parameters were obtained from STH-ObS or H/IPAH clinical databases. The demographics of UK participants are shown in Table 1. Participants were asked to wear/carry their devices consistently, and if possible, to wear their watch overnight. My Heart Counts data is securely transmitted to a centralised Synapse database. Upon consenting into the My Heart Counts study, both prospective and historic (multiple years—if stored by user on iCloud) HealthKit data were collected via the App. 21 patients had HealthKit available prior to their diagnosis date. The period of data available was a median of 10 months [Interquartile range 27, 42] prior to diagnosis.

### US validation cohort

Seventy-three participants from the US MHC Stanford Cohort were used as an external validation cohort, of which 28 self-reported to have PAH, 23 self-reported other cardiovascular diseases (used as diseased controls) and 22 self-reported to be healthy (Fig. S12). Demographics from individuals are listed in Table 5. The data distribution compared to the UK cohort and given in the Figs. S8–9. This cohort had significantly shorter data spans with median and IQRs of 2.0 [0.1, 6.0] years.

**Table 5:** US MHC participant demographics (external validation)

US	PH	Healthy	Disease control	Total
**Gender**				
Female	8	6	4	18
>Male	20	1	8	29
Unknown	0	15	11	26
**Ethnicity**				
White	22	12	16	50
Non-white	6	2	3	11
Unknown	0	8	4	12
**Age (years)**				
Median [min, max]	62 [23, 77]	42 [33, 71]	65 [37, 87]	62 [23, 87]
Unknown	0	15	11	26
**BMI**				
Underweight	2	0	20	22
Normal	1	1	1	3
Overweight or Obese	4	4	2	10
Unknown	21	17	0	38

Data are presented as median [min, max].

### Data processing

My Heart Counts Data was parsed with each user’s PseudoID to enable matching to their clinical data. HealthKit data (HealthKit features are italicised throughout the manuscript) were processed and cleaned to correct for duplicates, outliers (Table S13) and missing data prior to aggregated at hourly and daily levels. Exact duplicate records arising from software updates or queues in software pipelines (about 15% of the data) were removed. For each HealthKit variable, data outliers were regarded as values too high or too low to be achieved in a single day were removed (about 13.7% of the data). Missing data, including errors in the formatting of dates, or not-a-number values accounted for less than 0.01% of the data. No data imputation was performed; missing data were kept as missing. Aggregated daily data (see Table S14) were then linked to IPAH clinical phenotype data, questionnaire responses and uploaded to Google Cloud using a BigQuery data warehouse. For the machine learning classifier, we ran a featurization pipeline which includes macro-statistical features, frequency-domain stats, and ARIMA features to all variables at a monthly aggregation level by patient. The features were cc_lag0, cc_lag1, cc_lag2, N, MAX, MIN, P2P, RMS, STD, VAR, MEAN, PEAK, SKEW, MAX_f, POWER, SUM_f, VAR_f, ar.L1, ar.L2, MEAN_f, PEAK_f, SKEW_f, sigma2, KURTOSIS, KURTOSIS_f, CREST FACTOR.

### HealthKit and calculated variables measured by phone and watch

The following variables were directly acquired from HealthKit: Time *Awake* (hrs), *VO*_*2*_*Max* (mL O_2_/ kg/ min), *TimeInBed* (hrs), *averageHeartRate* (beats/min), *stepCount* (steps), Time *Asleep* (hrs), *appleStandTime* (hrs), *flightsClimbed*, *restingHeartRate* (beats/min), *basalEnergyBurned* (KCal), *activeEnergyBurned* (KCal), *distanceWalkingRunning* (m), *walkingHeartRate* Average (daily, beats/min), *heartRateVariabilitySDNN* (ms).

#### Walking/climbing pace (Gait speed)

Walking/climbing duration was calculated from the start and end time of each activity (seconds). Any duration of less than 0.5 s was excluded. To calculate pace, the step count or flights climbed was then divided by the duration.

***The following activity, heart rate and sleep metrics were calculated***:

1 $${\rm{HeartRateReserve}}={averageHeartRate}-{restingHeartRate}$$

2 $${\rm{CardiacEffort}}=\frac{{walkingHeartRate}}{\frac{{distanceWalkingRunning}}{{\rm{duration}}}}$$

3 $${\rm{StepCountPace}}=\left(\frac{{stepCount}}{{\rm{duration}}}\right){\rm{in\; a\; day}}$$

4 $${\rm{StepCountPaceMean}}={avg}\left(\frac{{stepCount}}{{\rm{duration}}}\right){\rm{in\; a\; day}}$$

5 $${\rm{StepCountPaceMax}}=\max \left(\frac{{stepCount}}{{\rm{duration}}}\right){\rm{in\; a\; day}}$$

6 $${\rm{FlightsClimbedPace}}=\left(\frac{{flightsClimbed}}{{\rm{duration}}}\right){\rm{in\; a\; day}}$$

7 $${\rm{FlightsClimbedPaceMean}}={avg}\left(\frac{{flightsClimbed}}{{\rm{duration}}}\right){\rm{in\; a\; day}}$$

8 $${\rm{FlightsClimbedPaceMax}}=\max \left(\frac{{flightsClimbed}}{{\rm{duration}}}\right){\rm{in\; a\; day}}$$

9 $${\rm{BedBound}}={inBed}\,{if}\,{inBed}\ge \frac{18{hrs}}{{\rm{day}}}$$

Pace over threshold of maximum effort (70%) is calculated where StepCountPace (Eq. (3)) or FlightsClimbedPace (Eq. (6)) is higher than 70% of maximum on any given day, and normalised to a continuous variable bound between 0 and 1, reflecting the proportion of time spent above 70% of their maximum effort in a day,

10 $${PaceHigher}70{pct}=\frac{{\sum }_{i}^{i\in {day}}{t}_{i}({P}_{i} > 0.7{P}_{\max })}{{\sum }_{i}^{i\in {day}}{t}_{i}({P}_{i} > 0)},$$

where ${t}_{i}$ is duration of measured interval, ${P}_{i}$ is the interval pace, and ${P}_{\max }$ is the overall maximum pace across all intervals.

The percentage of the data removed based is described in detail in Supplementary Table S13. Briefly, the lower bound was 3.0% (65,969 rows), the upper bound was 10.2% (216, 359 rows), and the illogical dates removed was 0.0% (2 rows).

The algorithm used to process the data and new computed metrics is provided in Supplemental Methods. For the assessment of distribution similarity, we used a Mann–Whitney U test and linear mixed effects model including all demographic information, time as fixed covariates, and the Group as random covariates, i.e. ‘value ~ age + bmi + gender + ethnicity + months + pre-diagnosis’. A one-way ANOVA test was used to assess differences between 6-month periods immediately pre- and post-diagnosis.

### Self-reported questionnaire analysis

Within the first 7-days following enrolment into the MHC study, participants were requested to complete several surveys, including PAR-Q (Tables S3, 4), Lifestyle Questionnaires including smoking, diet and sleep (Tables S3 and S5)^20^, Life Satisfaction Survey Activity (Tables S3 and S6), three mindset questionnaires, the AAMM^22^, illness mindset^23^ and exercise process mindset^24^ (Tables S3 and S7), and Risk perception Survey, as previously described^20^ (Tables S3 and S8). Although the questionnaires can be completed multiple times, most participants completed them only once. Analyses were restricted to responses collected during the initial 7-day period. Kruskal–Wallis tests were applied to variables with Likert scales to identify significant differences between the 3 categories of participants (IPAH, disease controls (DC), and healthy controls (HC)) and *P* values adjusted using FDR correction. Fisher’s exact test was applied for variables with binary responses, using simulated *p*-values in R (v 4.3.1). Post-hoc Dunn tests were carried out for question responses with a significant (<0.05) *p* value (Dunn.test v1.3.6). Polychoric correlation matrices were calculated using the *polycor* package in R (v0.8-1) for each of the three mindset questionnaires. To deal with missing values (for questionnaire data & FAMD analysis only) prior to factor analysis of mixed data (FAMD), samples with >50% missing values were removed. Missing values were then imputed using the *imputeFAMD* function from the *missMDA* (v1.19). The best number of components to use in the imputation process was estimated as 2 (utilising the *estim_ncpFAMD* function from the *missMDA* package). FAMD was then carried out using the *FactoMineR* package (v 2.11).

### Developing a classifier for IPAH

To train generate predictive models, we compared two machine learning algorithms: binary classification using XGBoost (*xgboost* = *=* 2.0.3) and linear SVM models (*scikit-learn* = *=* 1.5.0). We performed an internal five-fold cross-validation using UK data only, using a 70/30% train/test split at the participant level (no mixing of participants in groups). Hyperparameters were chosen by Bayesian optimisation. The values used for XGBoost were: reg alpha :2, reg lambda :5, scale pos weight: ${2}^{{\left(-1\right)}^{1-i}}$, learning rate: 0.07, max depth: 6, n estimators: 30.

Our choice of XGBoost and SVM was driven by their superior capacity to model complex, non-linear feature interactions within high-dimensional space. Unlike classic time series models, their tree-based (XGBoost) and margin-maximising (SVM) architectures effectively tolerate the sparsity and patchiness inherent to long-term patient-captured data, allowing us to generate robust predictions regardless of missing time steps. Our model selection was based on the weakest cross–validation, i.e. the most conservative to compare receiver operating characteristic (ROC) curves, F1, precision and recall as evaluation metrics. The thresholds were chosen based on optimised ROC AUCs and F1 scores at a 0.1 granularity.

To evaluate the model, we tested on two holdout sets: internal (UK data) and external (US data). For the external validation, we retrained the models on all UK data using the US as test (Fig. S7). Thresholds have been chosen to be maximal based on ROC AUCs and F1. Finally, we re-trained the models using a small subset of the US data (20%) to improve the efficiency of transfer learning, making sure to exclude the 20% segment from the test set, avoiding data leakage. We use the external validation cohort to characterise data drift in a real situation, much like it should be done in ML systems design. We implemented the Population Stability Index (PSI) to track any potential performance degradation. The PSI was calculated by first segmenting the data range of a single variable into a specified number of buckets. These buckets were equally sized bins across the value range. For each corresponding bucket, we extract the difference between the expected and actual population proportions, multiplies this difference by the natural logarithm of the ratio of the expected to actual proportion. The PSI for that variable is determined by summing these individual, per-bucket contributions, providing a single quantitative measure of the distribution’s shift between the two datasets. Finally, features importance was defined by Decision Tree Importance.

## Supplementary information

Supplementary Information

## Data Availability

The datasets generated and analysed during the current study are not publicly available due to the small sample size and rare disease origins of the research, and the potential to re-identify individual participants, but are available from the corresponding author for research purposes on reasonable request.

## Code availability

The code is available here: https://github.com/jadsm/myheartcounts_ph and the runtime requirements were of at least 8GB RAM & 2.4 GHz, although higher specs are recommended. Parallelization is recommended. The work was developed on an Apple Silicon M3 96GB running Python 3.11.7 using the virtual environment in the requirements.txt file. Visualisations are interactive and have been done in Altair (*altair* = *=5.3.0*) and deployed in Google App Engine (available at https://mhc-imperial.silico.science).
