# Supplementary material for: Assessing the feasibility of using smartphone data to identify risk of idiopathic pulmonary arterial hypertension
Source: NPJ Cardiovasc Health. 2026 Mar 25;3:16. doi: 10.1038/s44325-026-00114-9 (PMC13018193; doi:10.1038/s44325-026-00114-9)
Supplement: Supplementary file 1 — Supplementary Information [file 44325_2026_114_MOESM1_ESM.docx]

# **A pilot study assessing the feasibility of physical activity data to detect pulmonary arterial hypertension**

# Delgado-SanMartin et al.

## **Supplementary Material**

1. Supplementary Figures S1-S12
2. Supplementary Tables S1-S14
3. **Supplementary Figures**

*
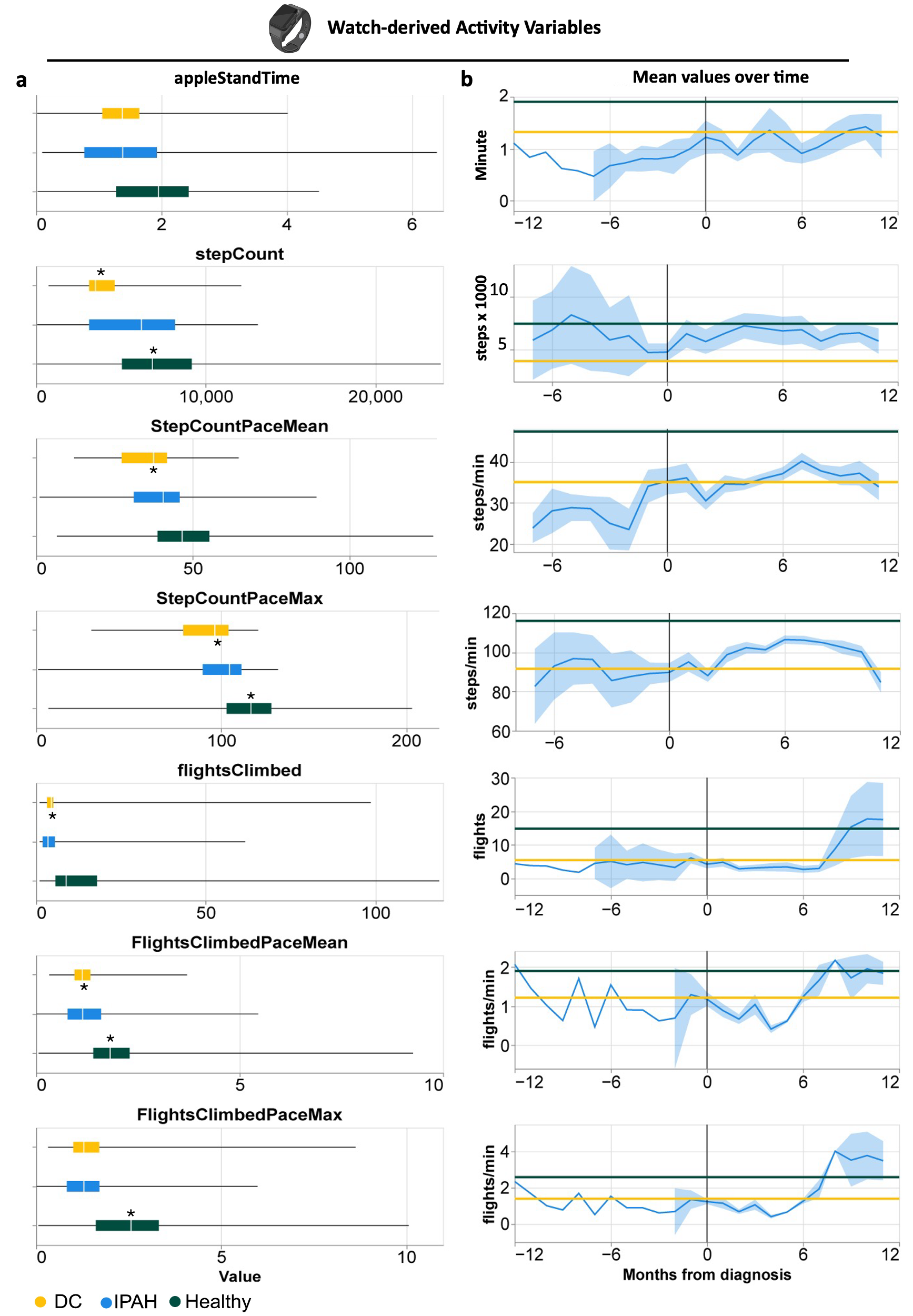
*

**Figure S1: Watch accelerometer metrics for UK cohort.**

**a.**Box plots showing the distribution of watch-derived physical activity metrics in IPAH cases prior to diagnosis. **b.** Longitudinal trends in monthly mean values of the same activity metrics from 12 months before to 12 months after diagnosis (month 0), with shaded areas representing 95% confidence intervals. Lines for disease controls (DC) and healthy volunteers (Healthy) represent group averages across the entire 24-month period. Statistical comparisons between IPAH cases and controls were performed using the Mann–Whitney U test; p < 0.05 was considered significant.

*
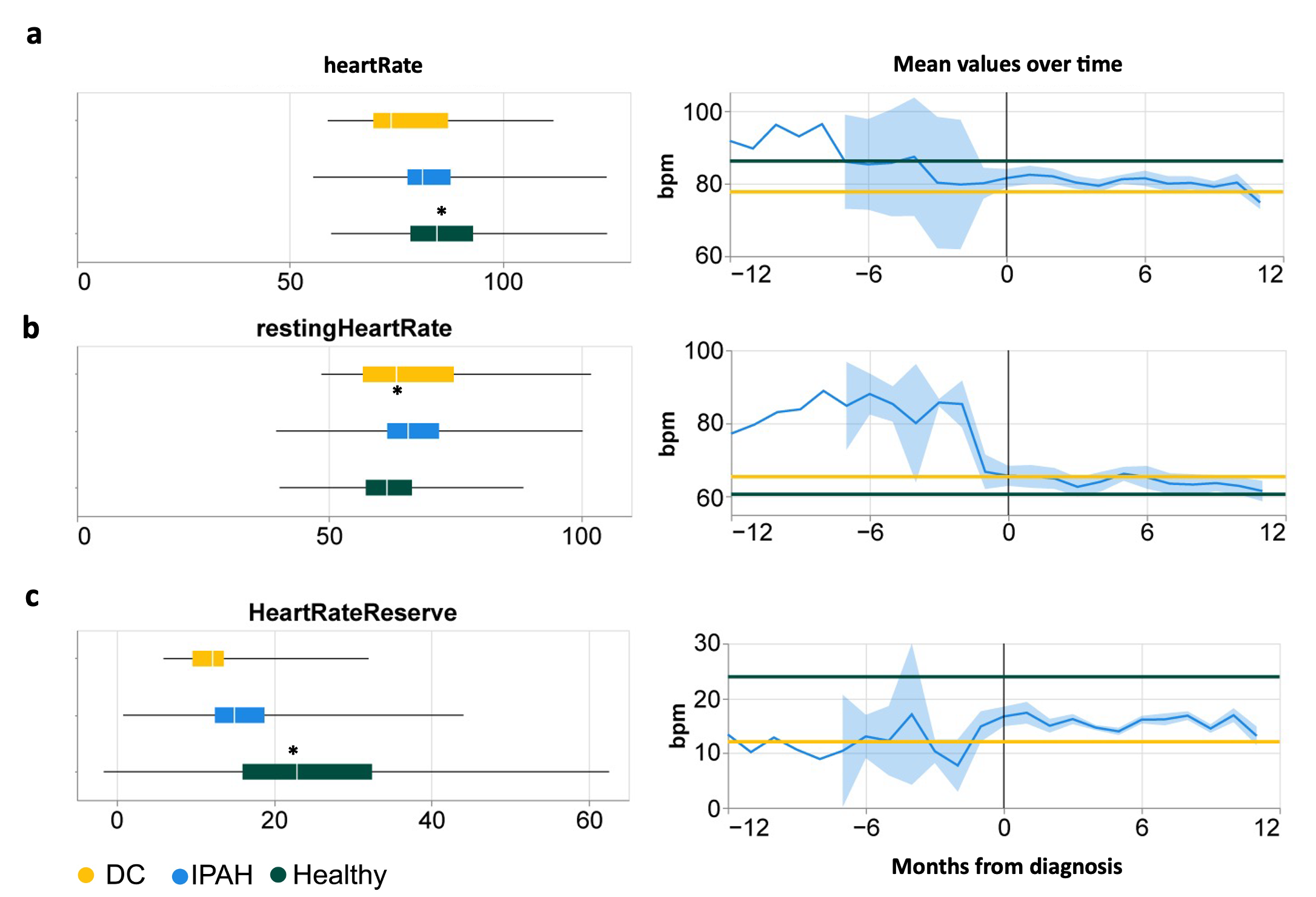
*

**Figure S2: Watch-derived heart rate metrics from UK cohort.**

Box plots (left) show distributions of **a.** average heart rate (*heartRate*), **b.** resting heart rate (*restingHeartRate*) and **c.** heart rate reserve (heartRateReserve) in IPAH cases prior to diagnosis. Line plots (right) display monthly mean values from 12 months before to 12 months after diagnosis, with shaded areas representing 95% confidence intervals.

Groups include IPAH patients (blue), disease controls (DC, yellow), and healthy volunteers (green). Statistical comparisons were performed using the Mann–Whitney U test; p < 0.05 was considered as significant.

*
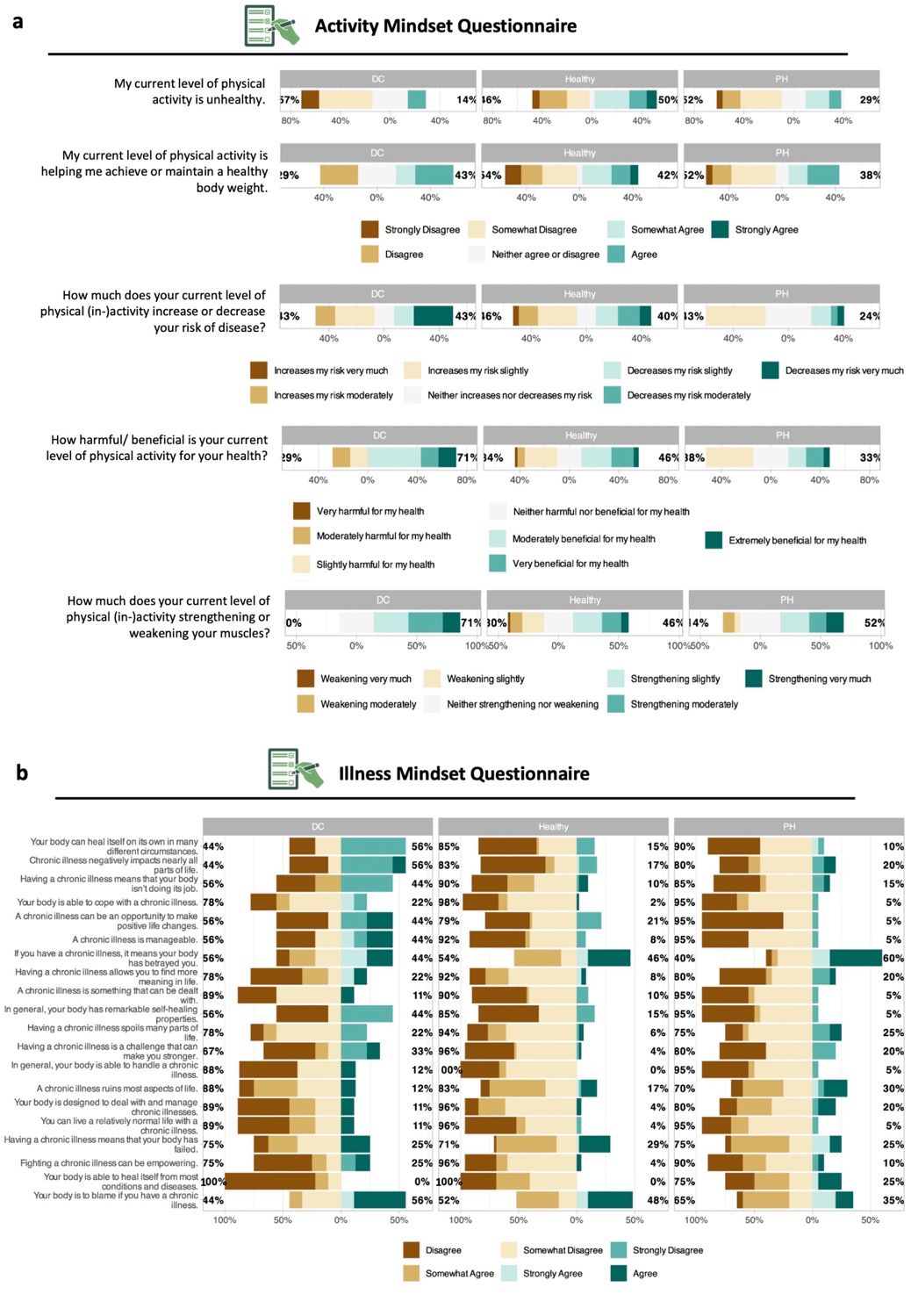
*

**Figure S3: Likert plots of responses to Activity and Illness Mindset questionnaires.** Colour scales are indicated under each group of questions with the positive and negative share of responses as a % for **a.** Activity Mindset Questions, and **b.** Illness mindset questions


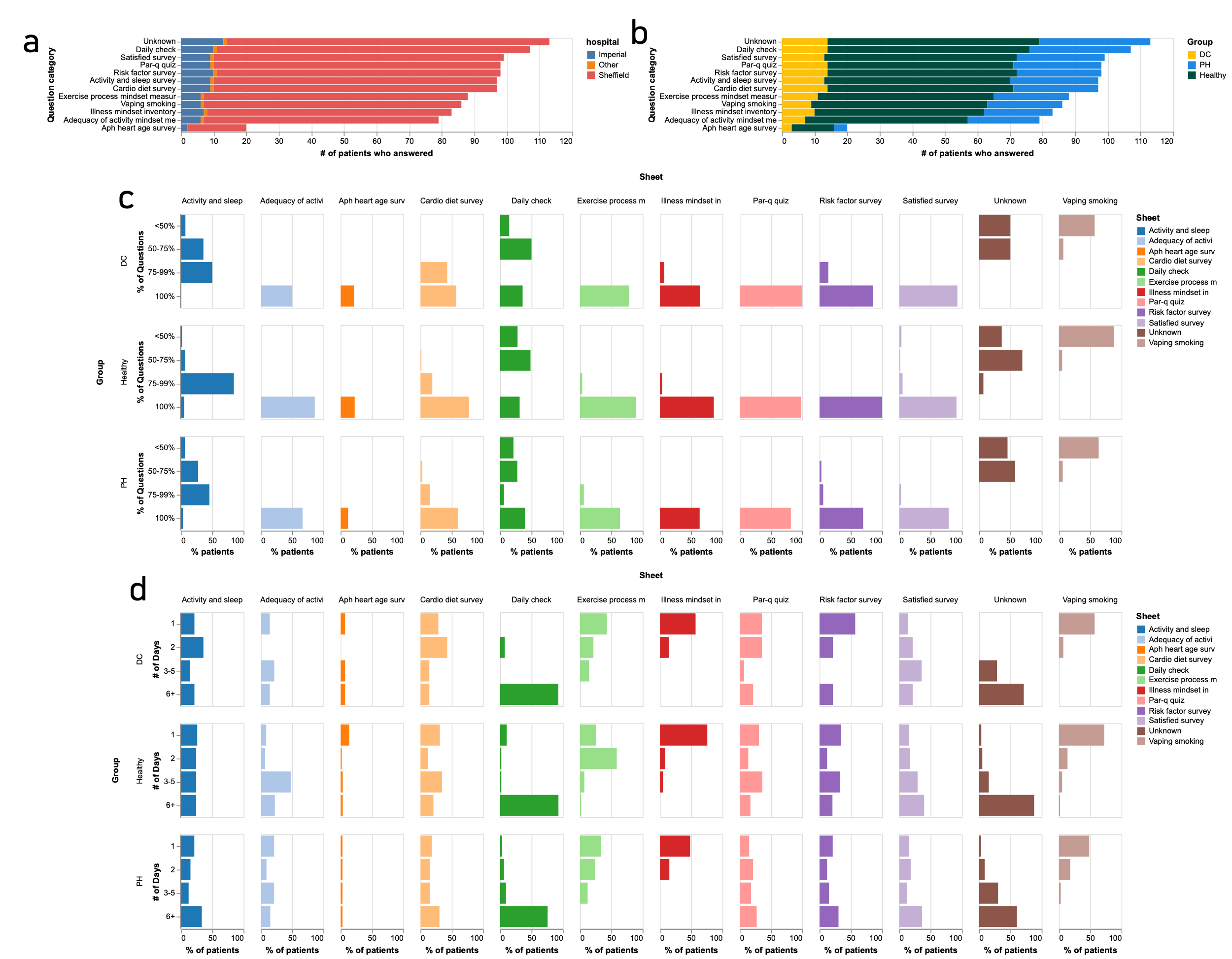


**Figure S4: Distribution of questionnaire answers within the My Heart Counts app.**

Number of participants that answered each questionnaire by **a.** hospital of origin (Imperial: blue, Sheffield: red, Other UK centre: Orange). **b.** disease group (Healthy: green, Disease-control: yellow, IPAH: blue). **c.** Percentage (%) of participants performing each questionnaire by disease group.


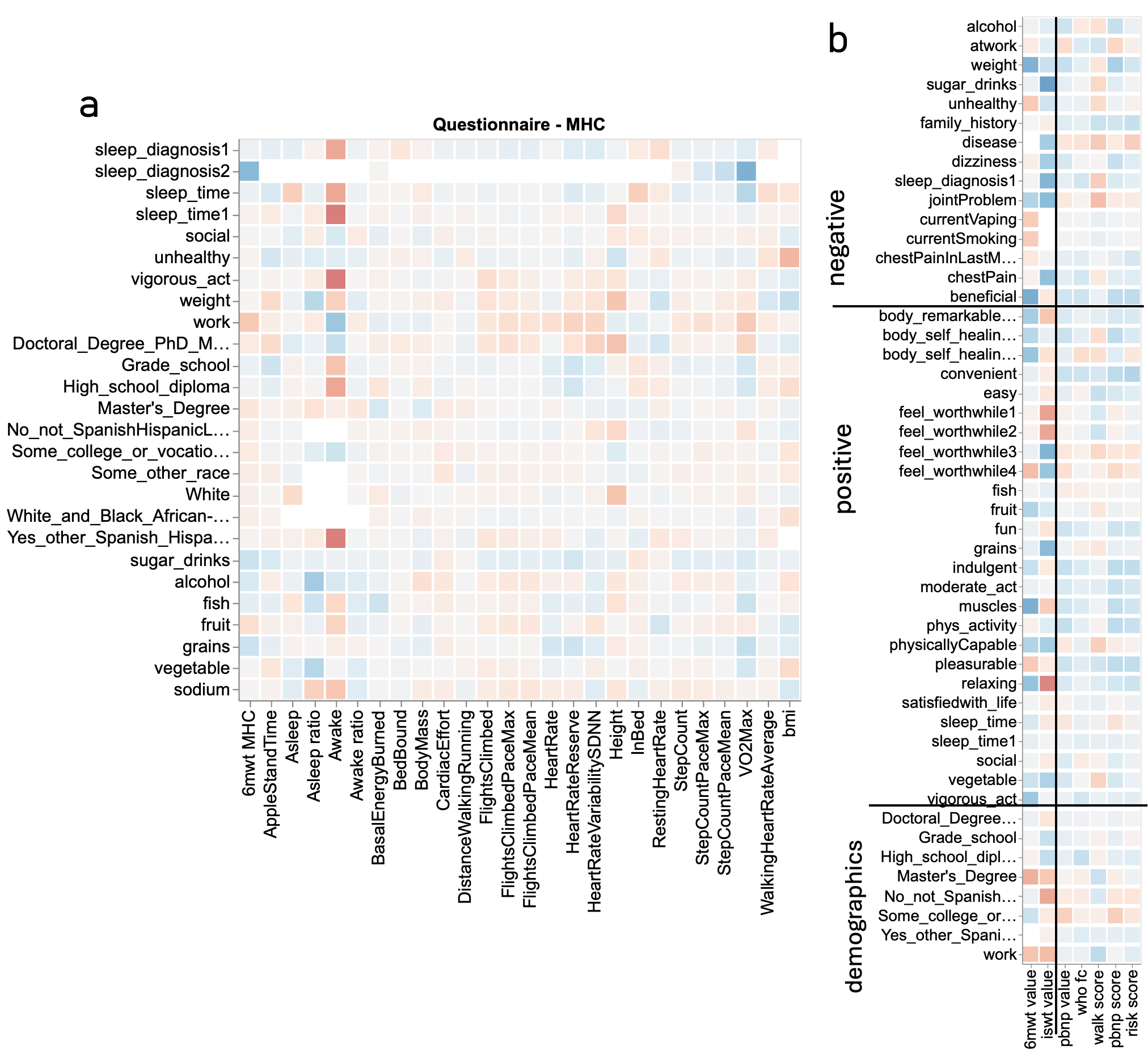


**Figure S5: *Heatmap showing P*earson’s correlations between *My Heart Counts and clinical* variables.** **a.** *correlation matrix* between watch and *p*hone *variables* and *My Heart Counts* questionnaire *responses*; and **b.** between clinical walk tests + risk scores *My Heart Counts* questionnaire *responses*. Colour intensity reflects the strength and direction of correlation (range: –1 to +1), with red indicating positive and blue indicating negative associations. p < 0.05 was considered significant, correlations $\rho>0.4.$ **b.** Correlation coefficients and p-values for 6-Min Walk Test (6MWT), Incremental Shuttle Walk Test (ISWT), and WHO Functional Class and ERS calculated walk scores (Walk score), NT-proBNP/BNP (pbnp score) and combined ERS.ESC Risk score (risk score).


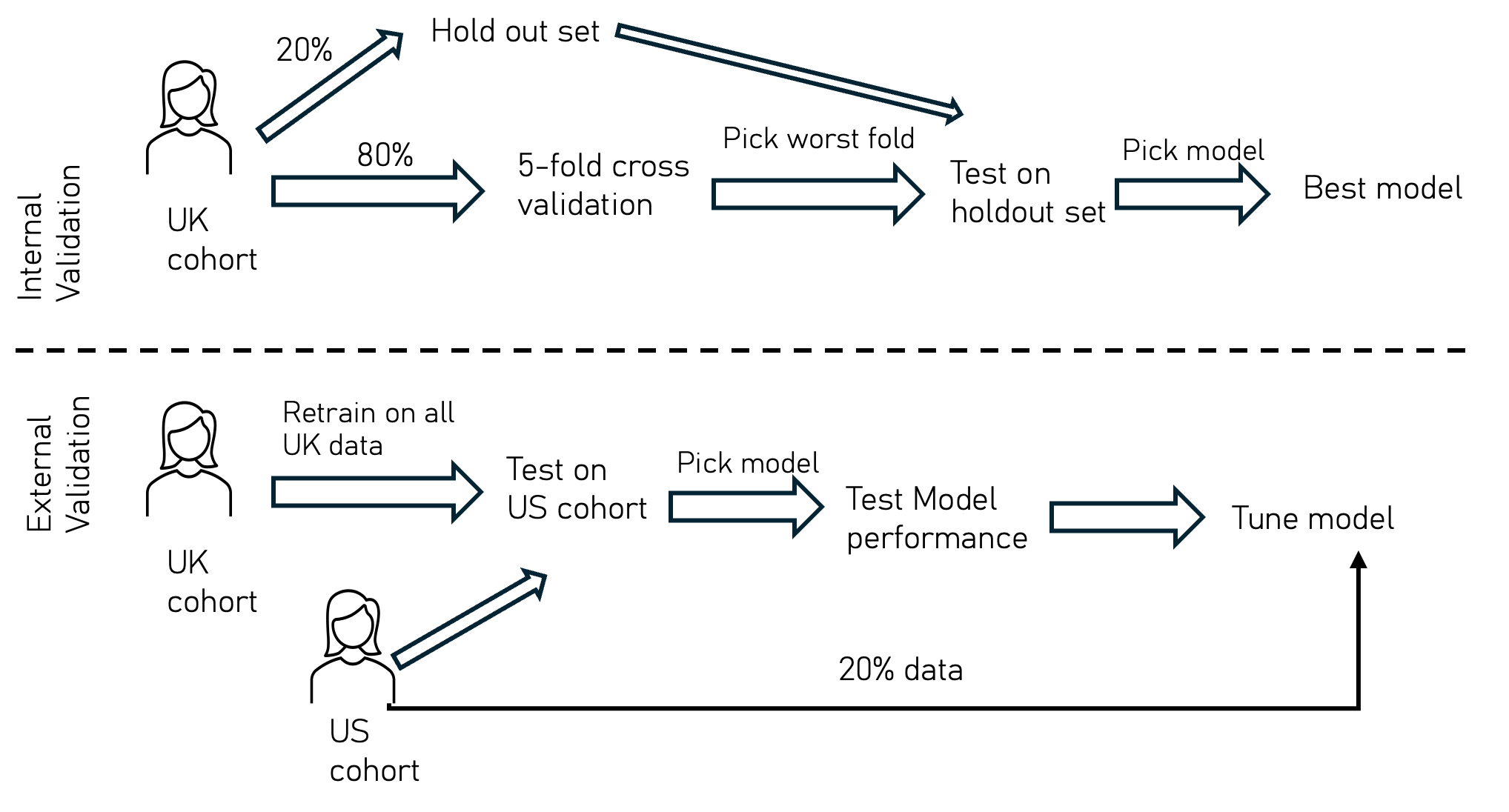


**Figure S6: Schematic overview of the validation steps for the internal (top) and external (bottom) validations.** For the internal validation 20% of data stratified by group (healthy, DC and IPAH) was hold out for testing. The remainder 80% was split into 5 sets of 70:30 train/test folds. The worst fold was then used to evaluate the holdout set. In the process hyperparameters were calibrated using Bayesian optimization. For the external validation the whole of the UK data was used to retrain a model with the optimal hyperparameters determined in the Internal validation. The model was evaluated using the US cohort providing poor results. To improve the results, 20% of the US cohort was added to the training data provided a ‘Retrained’ or ‘Tuned’ model. This was essential to achieve good translation of results.


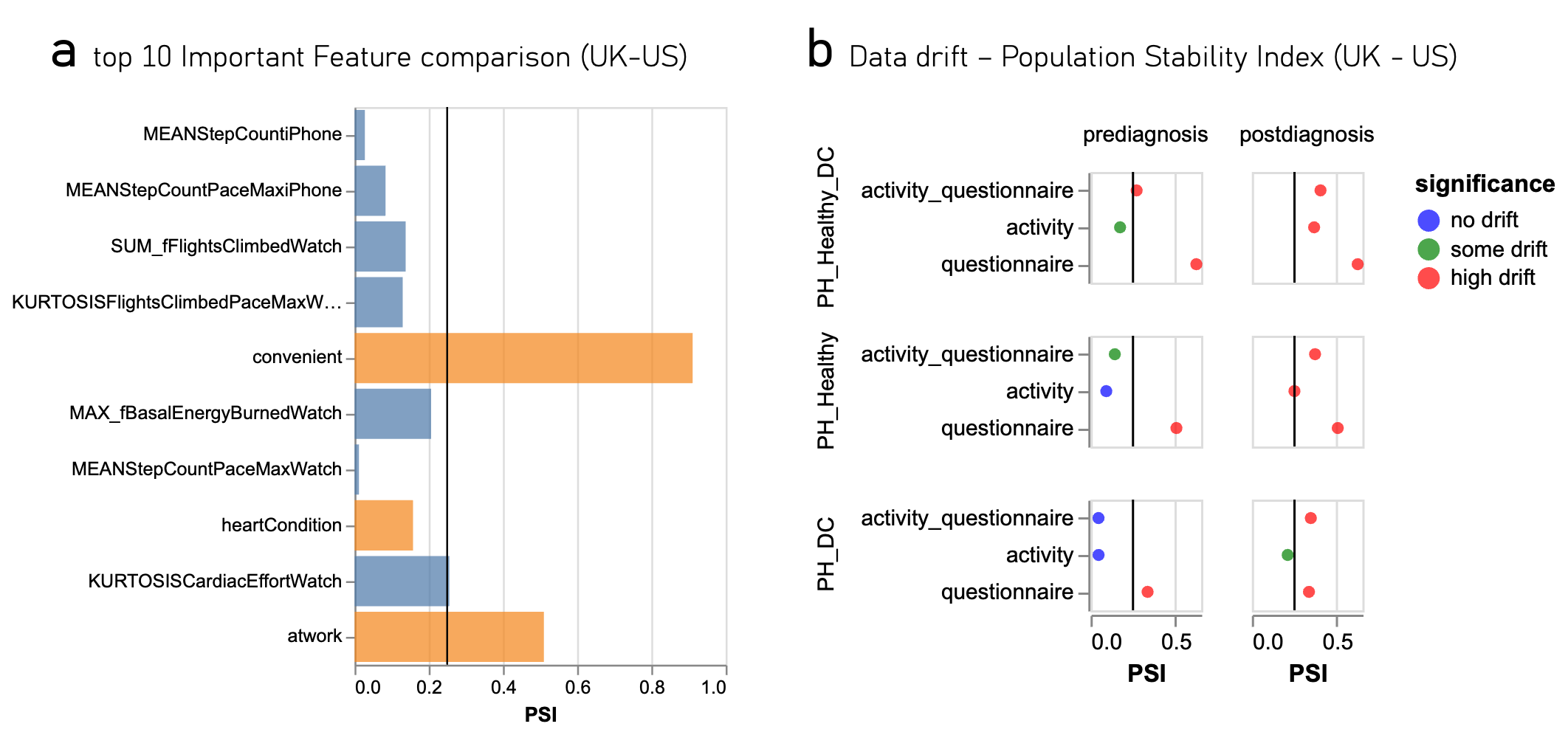


**Figure S7: Data drift by Population Stability Index**

**a.** Population Stability Index (PSI) for the top 10 variables. **b.** The data drift in distribution by PSI across all datasets.


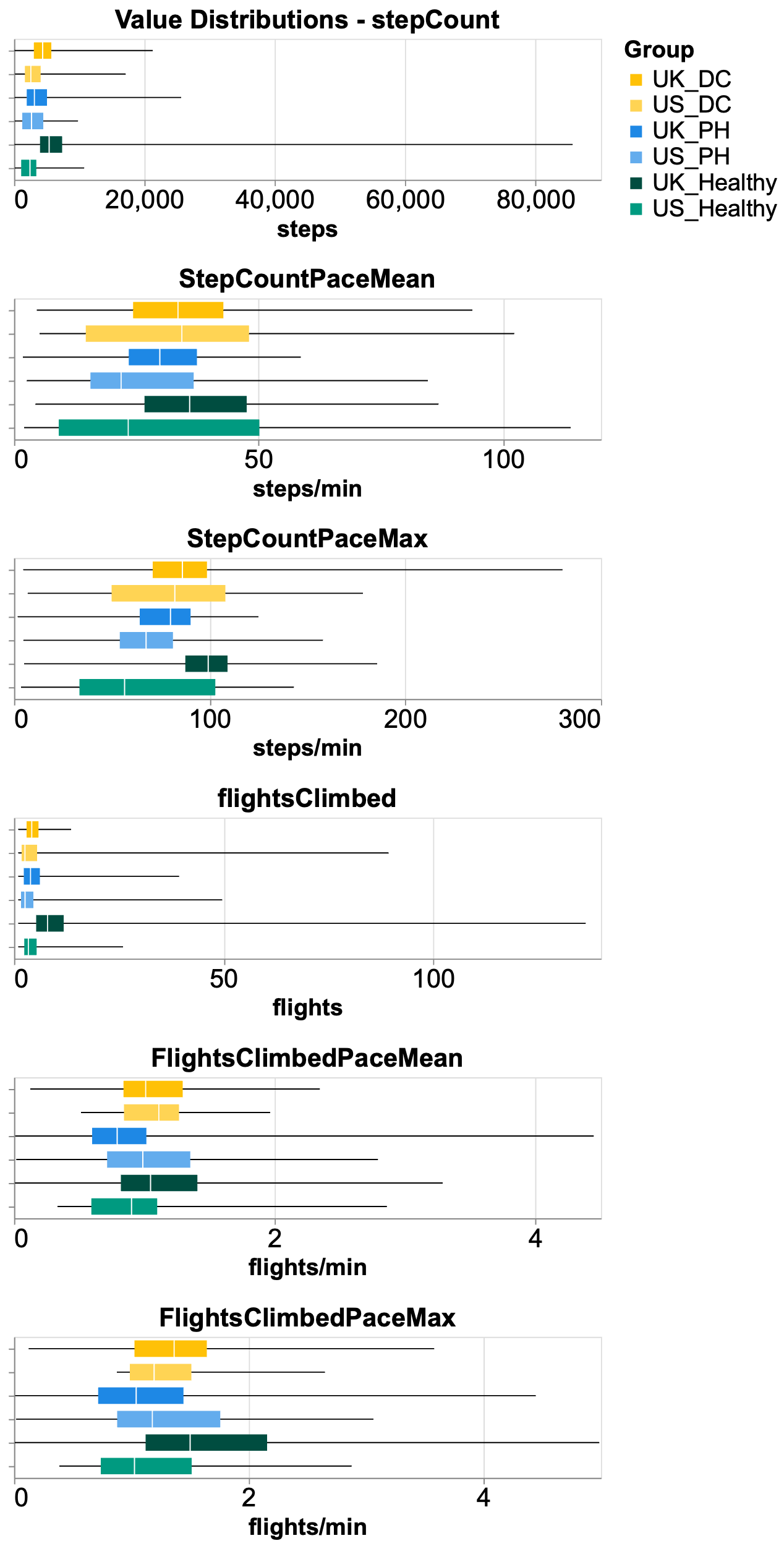


**Figure S8: Boxplots comparing UK and US distributions of iPhone metrics by group.** Box plots show distributions of variables. Groups include IPAH patients (blue), disease controls (DC, yellow), and healthy volunteers (green), the lighter hues are for the US cohort and darker for the UK.


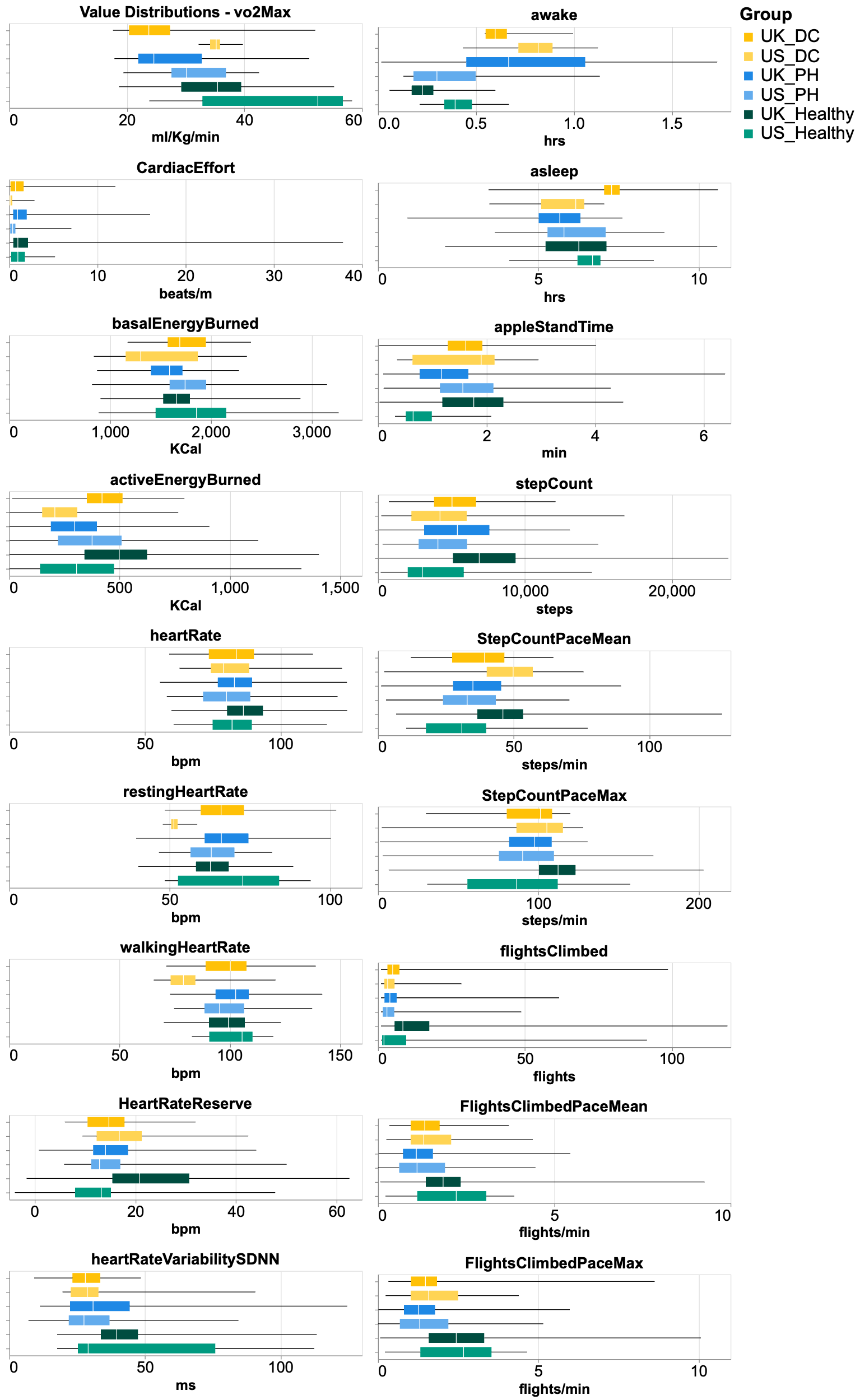


**Figure S9: Boxplots comparing UK and US distributions of Watch metrics by group.**

Box plots show distributions of variables. Groups include IPAH patients (blue), disease controls (DC, yellow), and healthy volunteers (green), the lighter hues are for the US cohort and darker for the UK.


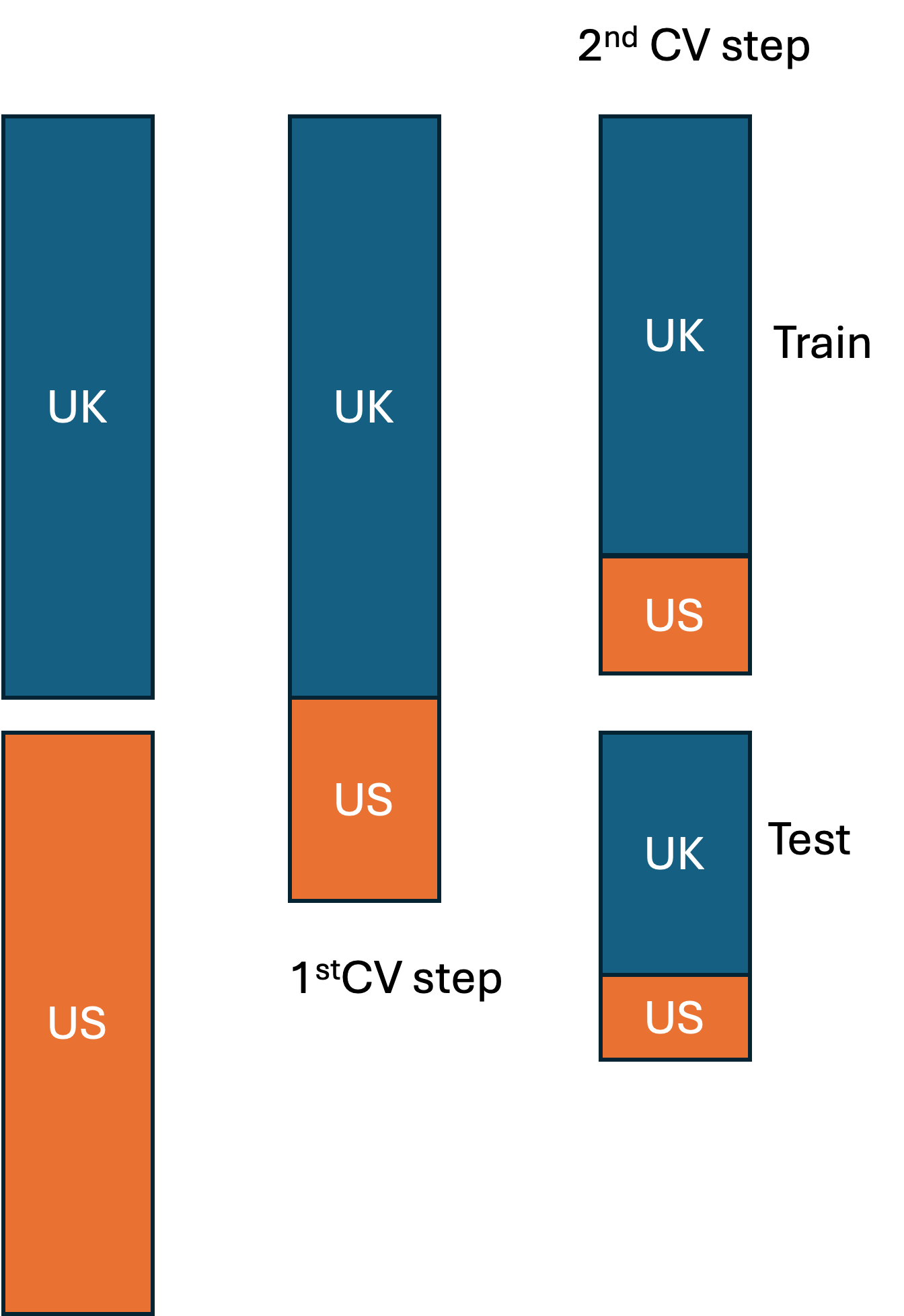


**Figure S10: Schematic for cross-validation strategy and retraining following dat transfer.** The strategy adopted for model retraining involved two rounds of cross-validations where the data was first separated into UK plus a random slice of 20%US cohort, validating on the rest. The initial training data was further split into training and validation at 80/20 ratio and rotated 5 times. We took the average performance of both cross-validations as the final output.


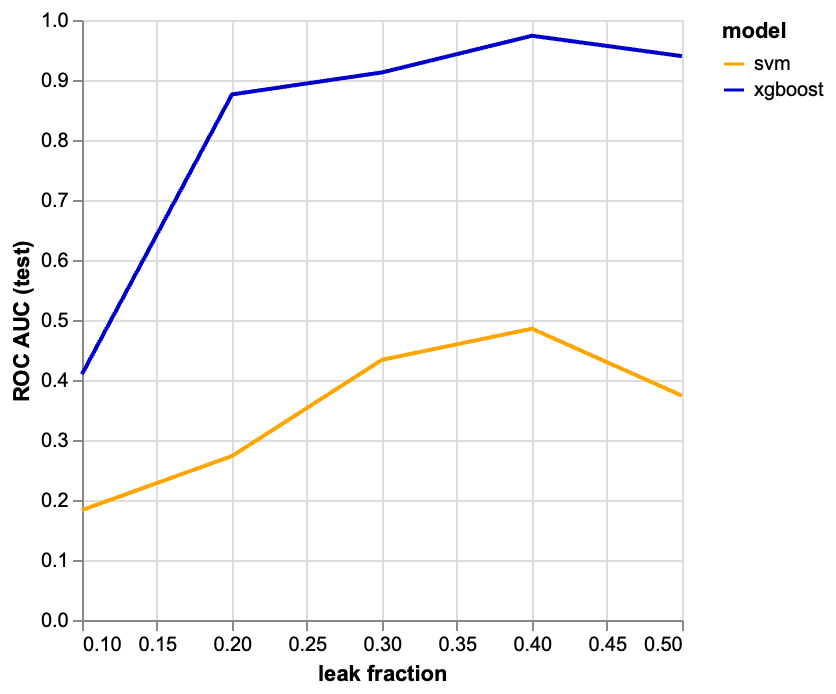


**Figure S11: ROC AUC for linear and xgboost model retraining at different ‘leak levels’.** The threshold of 20% was chosen as the minimum amount of data providing sufficient ROC AUC scores. This is dictated by the sharp increase from 10% to 20%.


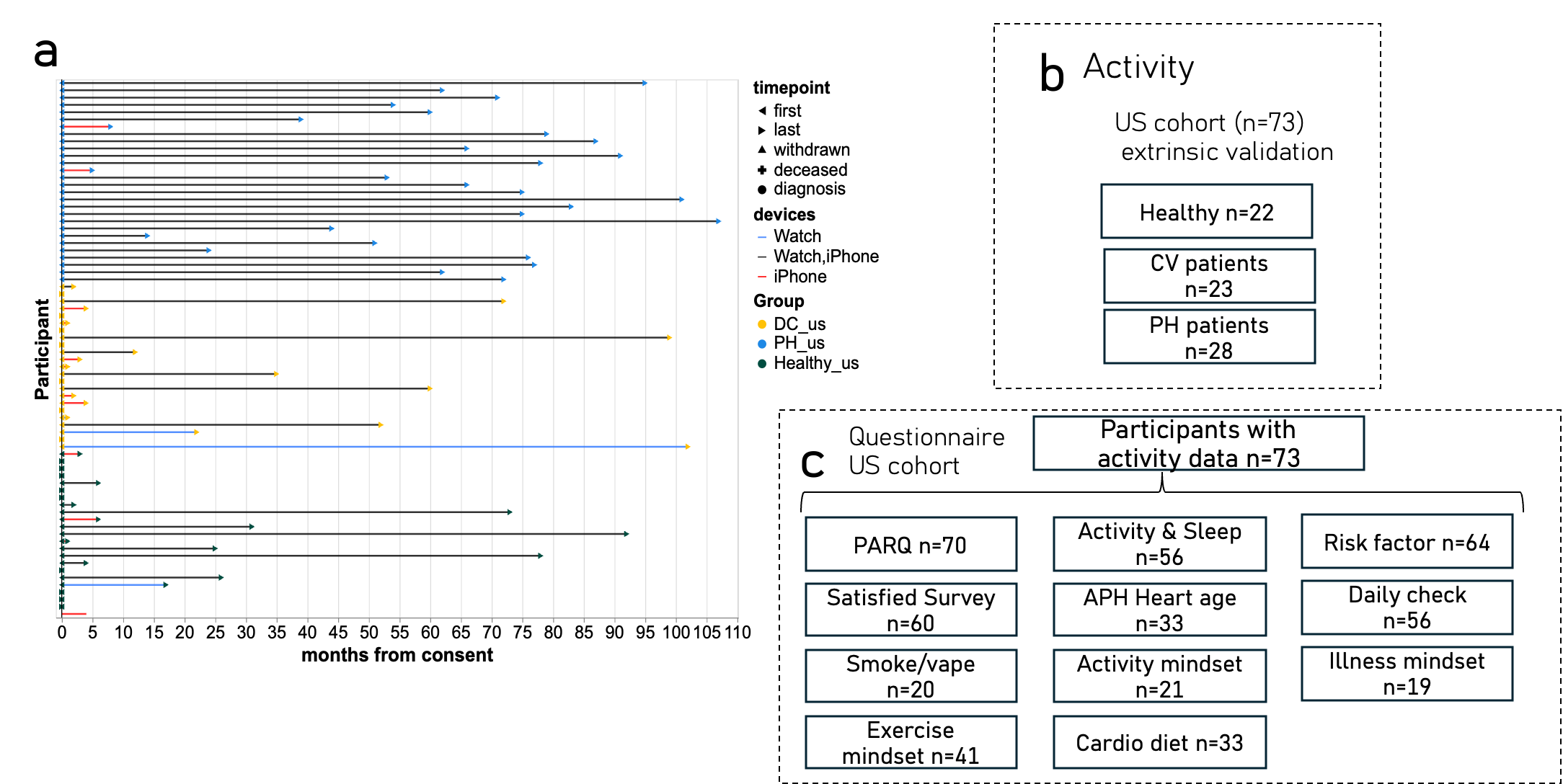


**Figure S12: Description of US cohort. a.** timeline of participants in relation to consent timeline. **b.** Participant counts for activity. **c.** Questionnaire answer availability with activity data.

## **Supplementary Tables**

**Table S1**: one-way anova values for means of each variable 6 months prior vs 6 months after diagnosis for IPAH group only. P-values <0.05 in green.

| **variable** | **device** | **p-value** |
| --- | --- | --- |
| StepCount | iPhone | 7.3E-25 |
| StepCountPaceMax | Watch | 7.4E-08 |
| StepCountPaceMax | iPhone | 5.8E-07 |
| FlightsClimbedPaceHigher70pct | iPhone | 1.3E-05 |
| FlightsClimbed | iPhone | 1.4E-04 |
| RestingHeartRate | Watch | 9.9E-04 |
| DistanceWalkingRunning | iPhone | 1.4E-03 |
| FlightsClimbedPaceMean | Watch | 3.1E-03 |
| StepCount | Watch | 3.3E-03 |
| FlightsClimbedPaceMax | Watch | 5.5E-03 |
| FlightsClimbed | Watch | 5.9E-03 |
| FlightsClimbedPaceMax | iPhone | 9.3E-03 |
| StepCountPaceHigher70pct | iPhone | 3.3E-02 |
| WalkingHeartRateAverage | Watch | 5.4E-02 |
| DistanceWalkingRunning | Watch | 8.3E-02 |
| StepCountPaceMean | Watch | 1.1E-01 |
| FlightsClimbedPaceHigher70pct | Watch | 2.2E-01 |
| ActiveEnergyBurned | Watch | 2.2E-01 |
| bmi | iPhone | 2.4E-01 |
| InBed | iPhone | 3.7E-01 |
| StepCountPaceHigher70pct | Watch | 4.1E-01 |
| HeartRateVariabilitySDNN | Watch | 4.5E-01 |
| StepCountPaceMean | iPhone | 5.9E-01 |
| FlightsClimbedPaceMean | iPhone | 6.0E-01 |
| BodyMass | iPhone | 6.4E-01 |
| HeartRate | Watch | 6.6E-01 |
| VO2Max | Watch | 6.8E-01 |
| HeartRateReserve | Watch | 7.5E-01 |
| BasalEnergyBurned | Watch | 7.8E-01 |
| AppleStandTime | Watch | 8.5E-01 |
| Height | iPhone | 8.7E-01 |
| CardiacEffort | Watch | 9.7E-01 |

**Table S2**: linear mixed effect p-values for seasonality granularities for selected features. Time granularities are Seasonally (hot, cold and shoulder months, spring/autumn), Weekly (weekdays or weekend), and Daily -divided into weekdays (day – 8am to 6pm / night) and weekends (work 9am to 5pm / evening 5pm to 10pm /night).

|  |  | Watch | | iPhone | |
| --- | --- | --- | --- | --- | --- |
|  |  | PH vs DC | PH vs Healthy | PH vs DC | PH vs Healthy |
| FlightsClimbedPaceMean | evenings |  |  | 2.28E-01 | 5.63E-05 |
|  | hot |  |  | 7.05E-04 | 2.22E-01 |
|  | night |  |  | 3.36E-01 | 2.82E-01 |
|  | shoulder |  |  | 3.58E-02 | 4.19E-01 |
|  | weekend |  |  | 5.11E-01 | 1.55E-29 |
|  | working |  |  | 1.69E-01 | 5.81E-01 |
| HeartRateVariabilitySDNN | evenings | 1.20E-01 | 2.18E-01 |  |  |
|  | hot | 6.12E-03 | 8.70E-02 |  |  |
|  | night | 1.49E-01 | 2.16E-01 |  |  |
|  | shoulder | 6.91E-02 | 3.40E-01 |  |  |
|  | weekend | 4.73E-01 | 2.15E-01 |  |  |
|  | working | 5.00E-02 | 2.40E-04 |  |  |
| RestingHeartRate | evenings | 9.77E-01 | 3.53E-01 |  |  |
|  | hot | 2.38E-01 | 7.43E-01 |  |  |
|  | night | 1.44E-02 | 5.55E-01 |  |  |
|  | shoulder | 4.95E-01 | 5.33E-02 |  |  |
|  | weekend | 1.42E-01 | 3.28E-03 |  |  |
|  | working | 1.80E-03 | 1.96E-01 |  |  |
| StepCountPaceMean | evenings |  |  | 1.45E-04 | 4.26E-08 |
|  | hot |  |  | 2.92E-03 | 4.90E-03 |
|  | night |  |  | 2.43E-01 | 1.07E-01 |
|  | shoulder |  |  | 2.81E-04 | 4.52E-03 |
|  | weekend |  |  | 2.01E-04 | 3.95E-21 |
|  | working |  |  | 2.22E-03 | 6.51E-09 |

***Table S3****: count of patients, questions and timepoints by questionnaire Sheet*

|  | **UK** | | | **US** | | |
| --- | --- | --- | --- | --- | --- | --- |
| **Sheet** | **patients** | **questions** | **timepoints** | **patients** | **questions** | **timepoints** |
| Vaping_Smoking | 88 | 12 | 37 | 21 | 10 | 25 |
| ACTIVITY AND SLEEP SURVEY | 112 | 9 | 1465 | 62 | 9 | 3451 |
| Illness_mindset_inventory | 85 | 20 | 34 | 20 | 20 | 20 |
| RISK FACTOR SURVEY | 101 | 7 | 205 | 93 | 7 | 175 |
| Exercise_process_mindset_measur | 90 | 7 | 61 | 22 | 7 | 37 |
| PAR-Q QUIZ | 100 | 7 | 205 | 137 | 7 | 175 |
| Adequacy_of_activity_mindset_me | 80 | 5 | 112 | 22 | 5 | 84 |
| DEMOGRAPHICS | 116 | 3 | 90 | 32 | 3 | 48 |
| CARDIO DIET SURVEY | 101 | 6 | 206 | 34 | 6 | 156 |
| DAILY CHECK | 111 | 10 | 1465 | 62 | 10 | 3451 |
| DAY ONE | 94 | 2 | 40 | 139 | 2 | 22 |
| SATISFIED SURVEY | 103 | 9 | 323 | 75 | 9 | 307 |
| APH HEART AGE SURVEY | 21 | 11 | 106 | 36 | 11 | 85 |

**Table S4**: PAR-Q QUIZ questions in questionnaire with exclusions

| **Question** | **exclude** | **Prompt** |
| --- | --- | --- |
| chestPain | FALSE | Do you feel pain in your chest when you do physical activity? |
| chestPainInLastMonth | FALSE | In the past month, have you had chest pain when you were not doing physical activity? |
| dizziness | FALSE | Do you lose your balanced because of dizziness or do you ever lose consciousness? |
| heartCondition | FALSE | has your doctor ever said that you have a heart condition and that you should only do physical activity recommended by a doctor? |
| jointProblem | FALSE | Do you have a bone or joint problem that could be made worse by a change in your physical activity? |
| physicallyCapable | FALSE | Do you know of any reason why you should not do physical activity? |
| prescriptionDrugs | TRUE | Is your doctor currently prescribing drugs (for example water pills) for your blood pressure or heart condition? |

**Table S5**: Lifestyle Questions in questionnaire with exclusions and groupings by Sheet (native to MHC)

| **Sheet** | **Question** | **exclude** | **Prompt** |
| --- | --- | --- | --- |
| Vaping_Smoking | currentSmokeless | FALSE | Do you currently use smokeless tobacco (chewing tobacco, snuff, snus, and dissolvable tobacco products)? |
| Vaping_Smoking | currentSmoking | FALSE | Do you current smoke cigarettes? |
| Vaping_Smoking | currentVaping | FALSE | Do you currently vape nicotine (use ‚Äúe-cigs,‚Äù ‚Äúmods,‚Äù ‚Äúvape pens,‚Äù ‚Äúvapes‚Äù, ‚ÄúJUULs‚Äù, etc)? |
| Vaping_Smoking | everQuitSmokeless | FALSE | During the past 12 months, have you tried to stop chewing tobacco (chewing tobacco, snuff, snus, and dissolvable tobacco products)?) |
| Vaping_Smoking | everQuitSmoking | FALSE | During the past 12 months, have you tried to stop smoking cigarettes? |
| Vaping_Smoking | everQuitVaping | FALSE | During the past 12 months, have you tried to stop vaping? |
| Vaping_Smoking | onsetSmoking | FALSE | How old were you when you smoked your first tobacco cigarette (in years)? |
| Vaping_Smoking | onsetVaping | FALSE | How old were you when you first vaped (in years)? |
| Vaping_Smoking | pastSmokeless | FALSE | Have you used smokeless tobacco (chewing tobacco, snuff, snus, and dissolvable tobacco products) in the past? |
| Vaping_Smoking | pastVaping | FALSE | Have you vaped in the past? |
| Vaping_Smoking | readinessQuitSmokeless | FALSE | On a scale of 1-10, how ready do you feel to quit smokeless tobacco? |
| Vaping_Smoking | readinessQuitSmoking | FALSE | On a scale of 1-10, how ready do you feel to quit smoking cigarettes? |
| Vaping_Smoking | readinessQuitVaping | FALSE | On a scale of 1-10, how ready do you feel to quit vaping |
| CARDIO DIET SURVEY | alcohol | FALSE | How many units of alcohol do you have per week? |
| CARDIO DIET SURVEY | fish | FALSE | How many servings of fish do you eat on an average week? |
| CARDIO DIET SURVEY | fruit | FALSE | How many cups of fruit do you eat in an average day? |
| CARDIO DIET SURVEY | grains | FALSE | How many servings of whole grains do you eat on an average day? |
| CARDIO DIET SURVEY | sugar_drinks | FALSE | How many beverages with added sugar do you drink every week? |
| CARDIO DIET SURVEY | vegetable | FALSE | How many cups of vegetables do you eat in an average day? |
| ACTIVITY AND SLEEP SURVEY | atwork | FALSE | Work Time Activity. |
| ACTIVITY AND SLEEP SURVEY | moderate_act | FALSE | Overall, how many minutes of moderate activity do you get in a week? |
| ACTIVITY AND SLEEP SURVEY | phys_activity | FALSE | Leisure Time Activity. |
| ACTIVITY AND SLEEP SURVEY | sleep_diagnosis1 | FALSE | Have you ever been told by a doctor or other health professional that you have a sleep disorder? |
| ACTIVITY AND SLEEP SURVEY | sleep_time | FALSE | How many hours of sleep did you get last night? |
| ACTIVITY AND SLEEP SURVEY | sleep_time | FALSE | How much sleep do think you need every night to be rested? (in hours) |
| ACTIVITY AND SLEEP SURVEY | sleep_time1 | FALSE | How much sleep do you usually get at night on weekdays or workdays? |
| ACTIVITY AND SLEEP SURVEY | vigorous_act | FALSE | Overall, how many minutes of vigorous activity do you get in a week? |
| ACTIVITY AND SLEEP SURVEY | work | FALSE | Do you do regular work? |

**Table S6**: Satisfied Survey Questions in questionnaire with exclusions

| **Question** | **exclude** | **Prompt** |
| --- | --- | --- |
| feel_worthwhile1 | FALSE | Overall, to what extent do you feel the things you do in your life are worthwhile? |
| feel_worthwhile2 | FALSE | How about happy? |
| feel_worthwhile3 | FALSE | How about worried? |
| feel_worthwhile4 | FALSE | How about depressed? |
| riskfactors1 | FALSE | Over the next 10 years how likely do you think it is that you personally will have a heart attack, stroke, or die due to cardiovascular disease? (choose one) |
| riskfactors2 | FALSE | Over the next 10 years, compared to others your age and sex, how would you rate your risk of having a heart attack, stroke, or dying due to cardiovascular disease? (choose one) |
| riskfactors3 | FALSE | Over your lifetime how likely do you think it is that you personally will have a heart attack, stroke, or die due to cardiovascular disease? (choose one) |
| riskfactors4 | FALSE | Over your lifetime, compared to others your age and sex, how would you rate your risk of having a heart attack, stroke, or dying due to cardiovascular disease? (choose one) |
| satisfiedwith_life | FALSE | Overall, how satisfied are you with life as a whole these days? |

**Table S7**: Mindset questions in questionnaire with exclusions and groupings by Sheet (native to MHC)

| **Sheet** | **Question** | **exclude** | **Prompt** |
| --- | --- | --- | --- |
| Illness_mindset_inventory | body_remarkable_self_healing | FALSE | In general, your body has remarkable self-healing properties. |
| Illness_mindset_inventory | body_self_healing_from_most_conditions_and_diseases | FALSE | Your body is able to heal itself from most conditions and diseases. |
| Illness_mindset_inventory | body_self_healing_in_many_different_circumstances | FALSE | Your body can heal itself on its own in many different circumstances. |
| Illness_mindset_inventory | chronic_illness_body_betrayal | FALSE | If you have a chronic illness, it means your body has betrayed you. |
| Illness_mindset_inventory | chronic_illness_body_blame | FALSE | Your body is to blame if you have a chronic illness. |
| Illness_mindset_inventory | chronic_illness_body_coping | FALSE | Your body is able to cope with a chronic illness. |
| Illness_mindset_inventory | chronic_illness_body_failure | FALSE | Having a chronic illness means that your body has failed. |
| Illness_mindset_inventory | chronic_illness_body_handling | FALSE | In general, your body is able to handle a chronic illness. |
| Illness_mindset_inventory | chronic_illness_body_management | FALSE | Your body is designed to deal with and manage chronic illnesses. |
| Illness_mindset_inventory | chronic_illness_body_meaning | FALSE | Having a chronic illness means that your body isn‚Äôt doing its job. |
| Illness_mindset_inventory | chronic_illness_challenge | FALSE | Having a chronic illness is a challenge that can make you stronger. |
| Illness_mindset_inventory | chronic_illness_empowering | FALSE | Fighting a chronic illness can be empowering. |
| Illness_mindset_inventory | chronic_illness_handling | FALSE | A chronic illness is something that can be dealt with. |
| Illness_mindset_inventory | chronic_illness_impact | FALSE | Chronic illness negatively impacts nearly all parts of life. |
| Illness_mindset_inventory | chronic_illness_management | FALSE | A chronic illness is manageable. |
| Illness_mindset_inventory | chronic_illness_more_meaning_in_life | FALSE | Having a chronic illness allows you to find more meaning in life. |
| Illness_mindset_inventory | chronic_illness_positive_opportunity | FALSE | A chronic illness can be an opportunity to make positive life changes. |
| Illness_mindset_inventory | chronic_illness_relatively_normal_life | FALSE | You can live a relatively normal life with a chronic illness. |
| Illness_mindset_inventory | chronic_illness_runing_life | FALSE | A chronic illness ruins most aspects of life. |
| Illness_mindset_inventory | chronic_illness_spoil | FALSE | Having a chronic illness spoils many parts of life. |
| Adequacy_of_activity_mindset_me | beneficial | FALSE | How harmful/beneficial is your current level of physical activity for your health? |
| Adequacy_of_activity_mindset_me | disease | FALSE | How much does your current level of physical (in-)activity increase or decrease your risk of disease? |
| Adequacy_of_activity_mindset_me | muscles | FALSE | How much is your current level of physical (in-)activity strengthening or weakening your muscles? |
| Adequacy_of_activity_mindset_me | unhealthy | FALSE | My current level of physical activity is unhealthy. |
| Adequacy_of_activity_mindset_me | weight | FALSE | My current level of physical activity is helping me achieve or maintain a healthy body weight. |
| Exercise_process_mindset_measur | convenient | FALSE | EXERCISING is: convenient |
| Exercise_process_mindset_measur | easy | FALSE | EXERCISING is: easy |
| Exercise_process_mindset_measur | fun | FALSE | EXERCISING is: fun |
| Exercise_process_mindset_measur | indulgent | FALSE | EXERCISING is: indulgent |
| Exercise_process_mindset_measur | pleasurable | FALSE | EXERCISING is: pleasurable |
| Exercise_process_mindset_measur | relaxing | FALSE | EXERCISING is: relaxing |
| Exercise_process_mindset_measur | social | FALSE | EXERCISING is: social |

**Table S8**: Risk Factor questions in questionnaire with exclusions and groupings by Sheet (native to MHC)

| **Sheet** | **Question** | **exclude** | **Prompt** |
| --- | --- | --- | --- |
| RISK FACTOR SURVEY | family_history | FALSE | Do you have a family history of early heart disease? |
| RISK FACTOR SURVEY | medications_to_treat | TRUE | Do you take medications to treat the following risk factors (indicate all that apply) |
| RISK FACTOR SURVEY | education_College_graduate_or_Baccalaureate_Degree | FALSE |  |
| RISK FACTOR SURVEY | education_Doctoral_Degree_PhD_MD_JD_etc | FALSE |  |
| RISK FACTOR SURVEY | education_Grade_school | FALSE |  |
| RISK FACTOR SURVEY | education_High_school_diploma | FALSE |  |
| RISK FACTOR SURVEY | education_Master's_Degree | FALSE |  |
| RISK FACTOR SURVEY | education_Some_college_or_vocational_school_or_Associate_Degree | FALSE |  |
| RISK FACTOR SURVEY | ethnicity_No_not_SpanishHispanicLatino | FALSE |  |
| RISK FACTOR SURVEY | ethnicity_Yes_Cuban | FALSE |  |
| RISK FACTOR SURVEY | ethnicity_Yes_other_Spanish_Hispanic_Latina | FALSE |  |
| RISK FACTOR SURVEY | heart_disease_Angina_heart_chest_pains | TRUE |  |
| RISK FACTOR SURVEY | heart_disease_Atrial_fibrillation_Afib | TRUE |  |
| RISK FACTOR SURVEY | heart_disease_Congenital_Heart | TRUE |  |
| RISK FACTOR SURVEY | heart_disease_Coronary_BlockageStenosis | TRUE |  |
| RISK FACTOR SURVEY | heart_disease_Coronary_StentAngioplasty | TRUE |  |
| RISK FACTOR SURVEY | heart_disease_Heart_AttackMyocardial_Infarction | TRUE |  |
| RISK FACTOR SURVEY | heart_disease_Heart_Bypass_Surgery | TRUE |  |
| RISK FACTOR SURVEY | heart_disease_Heart_Failure_or_CHF | TRUE |  |
| RISK FACTOR SURVEY | heart_disease_High_Coronary_Calcium_Score | TRUE |  |
| RISK FACTOR SURVEY | heart_disease_None_of_the_above | TRUE |  |
| RISK FACTOR SURVEY | heart_disease_Pulmonary_Hypertension | TRUE |  |
| RISK FACTOR SURVEY | heart_disease_nan | TRUE |  |
| RISK FACTOR SURVEY | race_Asian_Indian | FALSE |  |
| RISK FACTOR SURVEY | race_Black_African-American_or_Negro | FALSE |  |
| RISK FACTOR SURVEY | race_Chinise | FALSE |  |
| RISK FACTOR SURVEY | race_Filipino | FALSE |  |
| RISK FACTOR SURVEY | race_Some_other_race | FALSE |  |
| RISK FACTOR SURVEY | race_White | FALSE |  |
| RISK FACTOR SURVEY | race_White_and_Black_African-American_or_Negro_and_American_Indian | FALSE |  |
| RISK FACTOR SURVEY | race_White_and_Pacific_Islander | FALSE |  |
| RISK FACTOR SURVEY | vascular_PAH | TRUE |  |
| RISK FACTOR SURVEY | vascular_Abdominal_Aortic_Aneurysm | TRUE |  |
| RISK FACTOR SURVEY | vascular_Carotid_Artery_BlockageStenosis | TRUE |  |
| RISK FACTOR SURVEY | vascular_Carotid_Artery_Surgery_or_Stent | TRUE |  |
| RISK FACTOR SURVEY | vascular_None_of_the_above | TRUE |  |
| RISK FACTOR SURVEY | vascular_Peripheral_Vascular_Disease_BlockageStenosis_Surgery_or_Stent | TRUE |  |
| RISK FACTOR SURVEY | vascular_Stroke | TRUE |  |
| RISK FACTOR SURVEY | vascular_Transient_Ischemic_Attack_TIA | TRUE |  |
| RISK FACTOR SURVEY | vascular_nan | TRUE |  |
| DAILY CHECK | sleep_time | FALSE | How many hours of sleep did you get last night? |
| DAILY CHECK | sleep_time | FALSE | How much sleep do think you need every night to be rested? (in hours) |
| DEMOGRAPHICS | countryCode_CA | FALSE |  |
| DEMOGRAPHICS | countryCode_DE | FALSE |  |
| DEMOGRAPHICS | countryCode_GB | FALSE |  |
| DEMOGRAPHICS | countryCode_SE | FALSE |  |
| DEMOGRAPHICS | countryCode_SG | FALSE |  |
| DEMOGRAPHICS | countryCode_US | FALSE |  |
| DEMOGRAPHICS | patientFitzpatrickSkinType_Type_I | FALSE |  |
| DEMOGRAPHICS | patientFitzpatrickSkinType_Type_II | FALSE |  |
| DEMOGRAPHICS | patientBloodType_A+ | FALSE |  |
| DEMOGRAPHICS | patientBloodType_A- | FALSE |  |
| DEMOGRAPHICS | patientBloodType_O+ | FALSE |  |
| DEMOGRAPHICS | patientBloodType_O- | FALSE |  |

**Table S9**: Top questions with major differences between PH, Healthy and DC groups for the UK cohort assessed by Kruskal-Wallis test. Last column indicates the variables that have been excluded from the machine learning model (*).

| **Question ID** | **Category** | **Prompt** | **Log10 pvalues** |
| --- | --- | --- | --- |
| **PAH*** | VASCULAR | Which vascular disease diagnosis have you received? | -16.6 |
| **Pulmonary_Hypertension*** | HEART  DISEASE | Have you been diagnosed with any of the below diseases? | -13.3 |
| **prescriptionDrugs*** | PAR-Q QUIZ | Is your doctor currently prescribing drugs (for example water pills) for your blood pressure or heart condition? | -12.7 |
| **medications_to_treat*** | RISK FACTOR SURVEY | Do you take medications to treat the following risk factors (indicate all that apply) | -9.2 |
| **heartCondition*** | PAR-Q QUIZ | has your doctor ever said that you have a heart condition and that you should only do physical activity recommended by a doctor? | -8.9 |
| **work** | ACTIVITY AND SLEEP SURVEY | Do you do regular work? | -6.2 |
| **riskfactors2** | SATISFIED SURVEY | Over the next 10 years, compared to others your age and sex, how would you rate your risk of having a heart attack, stroke, or dying due to cardiovascular disease? (choose one) | -5.8 |
| **riskfactors4** | SATISFIED SURVEY | Over your lifetime, compared to others your age and sex, how would you rate your risk of having a heart attack, stroke, or dying due to cardiovascular disease? (choose one) | -4.2 |
| **physicallyCapable** | PAR-Q QUIZ | Do you know of any reason why you should not do physical activity? | -4.1 |
| **riskfactors1** | SATISFIED SURVEY | Over the next 10 years how likely do you think it is that you personally will have a heart attack, stroke, or die due to cardiovascular disease? (choose one) | -4 |
| **riskfactors3** | SATISFIED SURVEY | Over your lifetime how likely do you think it is that you personally will have a heart attack, stroke, or die due to cardiovascular disease? (choose one) | -3.2 |
| **Stroke*** | VASCULAR | Which vascular disease diagnosis have you received? | -3.1 |
| **sugar_drinks** | CARDIO DIET SURVEY | How many beverages with added sugar do you drink every week? | -2.7 |
| **Grade_school** | education | What is the highest grade in school you finished? Choose the best answer. | -2.5 |
| **Heart_Failure_or_CHF_and_Pulmonary_Hypertension*** | HEART  DISEASE | Have you been diagnosed with any of the below diseases? | -2.1 |
| **chronic_illness_more_meaning_in_life** | Illness  Mindset  inventory | Having a chronic illness allows you to find more meaning in life. | -2 |
| **relaxing** | Exercise  Process  Mindset | EXERCISING is: relaxing | -2 |
| **Atrial_fibrillation_Afib*** | HEART  DISEASE | Have you been diagnosed with any of the below diseases? | -1.8 |
| **fruit** | CARDIO DIET SURVEY | How many cups of fruit do you eat in an average day? | -1.7 |
| **body_self_healing_from_most_conditions_and_diseases** | Illness_mindset_inventory | Your body is able to heal itself from most conditions and diseases. | -1.7 |
| **body_self_healing_in_many_different_circumstances** | Illness_mindset_inventory | Your body can heal itself on its own in many different circumstances. | -1.7 |
| **Atrial_fibrillation_Afib_and_Pulmonary_Hypertension*** | HEART | Have you been diagnosed with any of the below diseases? | -1.5 |
|  | DISEASE |  |  |
| **grains** | CARDIO DIET SURVEY | How many servings of whole grains do you eat on an average day? | -1.5 |
| **chronic_illness_handling** | Illness_mindset_inventory | A chronic illness is something that can be dealt with. | -1.4 |

**Table S10**: significant Pearson’s correlation between Questionnaire and HealthKit variables and Risk Scores – includes pvalue >0.05 and correlations $\rho>0.4$

| **Variable origin** | **Variable** | **Risk scores** | **correlation** | **pvalue** | **n_patients** |
| --- | --- | --- | --- | --- | --- |
| HealthKit | FlightsClimbed | 6mwt_value | 0.5344 | 0.0020 | 8 |
| HealthKit | HeartRateReserve | 6mwt_value | 0.6312 | 0.0155 | 6 |
| HealthKit | HeartRateVariabilitySDNN | 6mwt_value | 0.6038 | 0.0222 | 6 |
| HealthKit | BasalEnergyBurned | 6mwt_value | -0.5294 | 0.0288 | 7 |
| HealthKit | FlightsClimbedPaceHigher70pct | 6mwt_value | -0.4204 | 0.0325 | 7 |
| HealthKit | StepCountPaceMean | 6mwt_value | 0.4204 | 0.0364 | 8 |
| HealthKit | RestingHeartRate | iswt_value | -0.8568 | 0.0001 | 8 |
| HealthKit | WalkingHeartRateAverage | iswt_value | -0.8522 | 0.0004 | 8 |
| HealthKit | StepCount | iswt_value | 0.5169 | 0.0041 | 13 |
| HealthKit | HeartRateVariabilitySDNN | iswt_value | 0.6598 | 0.0196 | 7 |
| HealthKit | HeartRateReserve | iswt_value | 0.5890 | 0.0267 | 8 |
| HealthKit | AppleStandTime | pbnp_score | -0.4434 | 0.0001 | 25 |
| HealthKit | AppleStandTime | pbnp_value | -0.4336 | 0.0001 | 25 |
| HealthKit | StepCount | walk_score | -0.5358 | <0.0001 | 21 |
| HealthKit | RestingHeartRate | walk_score | 0.6628 | 0.0001 | 14 |
| HealthKit | HeartRateReserve | walk_score | -0.6410 | 0.0002 | 14 |
| HealthKit | HeartRateVariabilitySDNN | walk_score | -0.6254 | 0.0006 | 13 |
| HealthKit | FlightsClimbed | walk_score | -0.4429 | 0.0023 | 17 |
| HealthKit | FlightsClimbedPaceHigher70pct | walk_score | 0.4896 | 0.0028 | 13 |
| HealthKit | VO2Max | walk_score | -0.6927 | 0.0060 | 7 |
| HealthKit | HeartRate | who_fc | -0.4107 | 0.0010 | 18 |
| Questionnaire | Master's_Degree | 6mwt_value | 0.5018 | <0.0001 | 10 |
| Questionnaire | beneficial | 6mwt_value | -0.7124 | <0.0001 | 5 |
| Questionnaire | muscles | 6mwt_value | -0.6944 | <0.0001 | 5 |
| Questionnaire | chronic_illness_body_meaning | 6mwt_value | 0.6129 | <0.0001 | 6 |
| Questionnaire | chronic_illness_impact | 6mwt_value | 0.6129 | <0.0001 | 6 |
| Questionnaire | jointProblem | 6mwt_value | -0.4413 | 0.0001 | 8 |
| Questionnaire | feel_worthwhile4 | 6mwt_value | 0.4372 | 0.0001 | 9 |
| Questionnaire | physicallyCapable | 6mwt_value | -0.4329 | 0.0001 | 8 |
| Questionnaire | relaxing | 6mwt_value | -0.5609 | 0.0002 | 5 |
| Questionnaire | body_self_healing_  in_many_different_circumstances | 6mwt_value | -0.5433 | 0.0002 | 6 |
| Questionnaire | fruit | 6mwt_value | -0.4421 | 0.0003 | 8 |
| Questionnaire | chronic_illness_body_management | 6mwt_value | -0.5334 | 0.0003 | 6 |
| Questionnaire | work | 6mwt_value | 0.4095 | 0.0008 | 8 |
| Questionnaire | chronic_illness_body_blame | 6mwt_value | 0.4931 | 0.0011 | 6 |
| Questionnaire | body_remarkable_self_healing | 6mwt_value | -0.4841 | 0.0013 | 6 |
| Questionnaire | chronic_illness_spoil | 6mwt_value | 0.4524 | 0.0030 | 6 |
| Questionnaire | chronic_illness_body_coping | 6mwt_value | 0.4492 | 0.0032 | 6 |
| Questionnaire | vigorous_act | 6mwt_value | -0.5245 | 0.0035 | 6 |
| Questionnaire | body_self_healing_  from_most_conditions_and_diseases | 6mwt_value | -0.4096 | 0.0078 | 6 |
| Questionnaire | sugar_drinks | iswt_value | -0.7957 | <0.0001 | 13 |
| Questionnaire | pastSmokeless | iswt_value | -0.7363 | 0.0001 | 10 |
| Questionnaire | relaxing | iswt_value | 0.7064 | 0.0002 | 12 |
| Questionnaire | feel_worthwhile3 | iswt_value | -0.6686 | 0.0002 | 13 |
| Questionnaire | sleep_diagnosis1 | iswt_value | -0.6551 | 0.0003 | 13 |
| Questionnaire | jointProblem | iswt_value | -0.6118 | 0.0007 | 14 |
| Questionnaire | grains | iswt_value | -0.6122 | 0.0009 | 13 |
| Questionnaire | HeartRate | iswt_value | -0.7210 | 0.0011 | 9 |
| Questionnaire | No_not_SpanishHispanicLatino | iswt_value | 0.5490 | 0.0017 | 16 |
| Questionnaire | chestPain | iswt_value | -0.5752 | 0.0017 | 14 |
| Questionnaire | feel_worthwhile1 | iswt_value | 0.5697 | 0.0024 | 13 |
| Questionnaire | chronic_illness_impact | iswt_value | 0.6532 | 0.0024 | 10 |
| Questionnaire | Cognitive_therapy__counselling | iswt_value | -0.5275 | 0.0027 | 16 |
| Questionnaire | feel_worthwhile2 | iswt_value | 0.5582 | 0.0030 | 13 |
| Questionnaire | feel_worthwhile4 | iswt_value | -0.5447 | 0.0040 | 13 |
| Questionnaire | chronic_illness_relatively_normal_life | iswt_value | -0.5897 | 0.0079 | 10 |
| Questionnaire | dizziness | iswt_value | -0.4886 | 0.0097 | 14 |
| Questionnaire | physicallyCapable | iswt_value | -0.4794 | 0.0114 | 14 |
| Questionnaire | riskfactors1 | iswt_value | -0.4603 | 0.0180 | 13 |
| Questionnaire | work | iswt_value | 0.4457 | 0.0198 | 14 |
| Questionnaire | College_graduate_  or_Baccalaureate_Degree | iswt_value | 0.4178 | 0.0216 | 16 |
| Questionnaire | vegetable | iswt_value | -0.4454 | 0.0226 | 13 |
| Questionnaire | chronic_illness_spoil | iswt_value | 0.5138 | 0.0244 | 10 |
| Questionnaire | chronic_illness_body_blame | pbnp_score | -0.5051 | <0.0001 | 25 |
| Questionnaire | chronic_illness_body_failure | pbnp_score | -0.4558 | <0.0001 | 24 |
| Questionnaire | chronic_illness_body_betrayal | pbnp_score | -0.4244 | <0.0001 | 25 |
| Questionnaire | chronic_illness_management | pbnp_score | -0.4241 | <0.0001 | 25 |
| Questionnaire | chronic_illness_body_blame | pbnp_value | -0.4044 | <0.0001 | 25 |
| Questionnaire | convenient | risk_score | -0.4294 | <0.0001 | 30 |
| Questionnaire | chronic_illness_positive_opportunity | risk_score | -0.4321 | <0.0001 | 27 |
| Questionnaire | chronic_illness_body_blame | risk_score | -0.4313 | <0.0001 | 27 |
| Questionnaire | chronic_illness_impact | walk_score | -0.6040 | <0.0001 | 16 |
| Questionnaire | Cognitive_therapy__counselling | walk_score | 0.4289 | <0.0001 | 26 |
| Questionnaire | jointProblem | walk_score | 0.4364 | <0.0001 | 22 |
| Questionnaire | chronic_illness_spoil | walk_score | -0.5229 | <0.0001 | 16 |
| Questionnaire | chronic_illness_body_meaning | walk_score | -0.4629 | 0.0002 | 16 |
| Questionnaire | everQuitSmoking | walk_score | -0.7967 | 0.0033 | 5 |
| Questionnaire | chronic_illness_positive_opportunity | who_fc | -0.4487 | 0.0001 | 16 |
| Questionnaire | chronic_illness_empowering | who_fc | -0.4373 | 0.0001 | 16 |

**Table S11**: patient numbers for each activity feature set. * are not sufficient to fit the models.

|  | iPhone | Watch |
| --- | --- | --- |
| All | | |
| Activity | 104 | 89 |
| Heart Rates | 0 | 92 |
| Fitness | 11 | 94 |
| Sleep | 0 | 34* |
| Prediagnosis | | |
| Activity | 91 | 70 |
| Heart Rates | 0 | 73 |
| Fitness | 3 | 75 |
| Sleep | 0 | 26* |

**Table S12**: decision tree cumulative feature importance for final XGboost model.*metrics are the ones related to walking or climbing stairs.

| Features | Cumulative importance | Questionnaire Prompt |
| --- | --- | --- |
| MEANStepCountiPhone* | 0.17 |  |
| MEANStepCountPaceMaxiPhone* | 0.31 |  |
| SUM_fFlightsClimbedWatch* | 0.40 |  |
| KURTOSISFlightsClimbedPaceMaxWatch* | 0.48 |  |
| Convenient | 0.55 | EXERCISING is: convenient |
| MAX_fBasalEnergyBurnedWatch | 0.61 |  |
| MEANStepCountPaceMaxWatch* | 0.66 |  |
| heartCondition | 0.71 | has your doctor ever said that you have a heart condition and that you should only do physical activity recommended by a doctor? |
| KURTOSISCardiacEffortWatch | 0.75 |  |
| Atwork | 0.80 | Work Time Activity. |
| riskfactors2 | 0.83 | Over the next 10 years, compared to others your age and sex, how would you rate your risk of having a heart attack, stroke, or dying due to cardiovascular disease? (choose one) |
| currentSmoking | 0.86 | Do you currently smoke? |
| riskfactors1 | 0.88 | Over the next 10 years how likely do you think it is that you personally will have a heart attack, stroke, or die due to cardiovascular disease? (choose one) |
| CREST FACTORHeightiPhone | 0.90 |  |
| KURTOSISStepCountPaceMeaniPhone* | 0.91 |  |
| STDAppleStandTimeWatch | 0.92 |  |
| MINStepCountPaceMaxiPhone* | 0.93 |  |
| MAXFlightsClimbedPaceMaxWatch* | 0.94 |  |
| MINFlightsClimbediPhone* | 0.95 |  |
| graduate_school | 0.96 | What is the highest grade in school you finished? Choose the best answer. |
| sigma2StepCountPaceHigher70pctiPhone* | 0.97 |  |
| feel_worthwhile2 | 0.98 | How about happy? |
| MAXDistanceWalkingRunningiPhone* | 0.99 |  |
| CREST FACTORFlightsClimbedPaceMaxiPhone* | 1.00 |  |
| Fish | 1.00 | How many servings of fish do you eat on an average week? |
| MINActiveEnergyBurnedWatch | 1.00 |  |
| ar.L1FlightsClimbedWatch* | 1.00 |  |

***Table S13****: bounds for each variable*

|  | **Lower bound** | **Upper bound** | **Units** | **Dropped rows (lower bound)** | **Dropped rows (upper bound)** |
| --- | --- | --- | --- | --- | --- |
| **stepCount** | 50 | 100000 | steps | 10321 | 232950 |
| **flightsClimbed** | 0 | 500 | flights |  | 179000 |
| **FlightsClimbedPaceMax** | 0 | 10000 | flights / min |  | 74073 |
| **FlightsClimbedPaceMean** | 0 | 10000 | flights / min |  | 74073 |
| **StepCountPaceMax** | 0 | 10000 | flights / min |  | 224716 |
| **StepCountPaceMean** | 0 | 10000 | flights / min |  | 224717 |
| **basalEnergyBurned** | 800 | 5000 | Kcal | 53538 | 153615 |
| **activeEnergyBurned** | 0 | 5000 | Kcal |  | 147805 |
| **appleStandTime** | 0 | 24 | hours |  | 91050 |
| **walkingHeartRateAverage** | 50 | 220 | beats / min | 137 | 71637 |
| **heartRate** | 30 | 220 | beats / min |  | 139638 |
| **restingHeartRate** | 30 | 220 | beats / min |  | 77725 |
| **heartRateVariabilitySDNN** | 0 | 150 | ms |  | 96884 |
| **vo2Max** | 0 | 60 | mL/Kg*min |  | 12279 |
| **Height** | 1.4 | 2.2 | m | 1 | 149 |
| **BodyMass** | 40 | 200 | Kg | 9 | 159 |
| **distanceWalkingRunning** | 0 | 60 | m |  | 17610 |
| **InBed** | 0 | 24 | Hours |  | 29688 |
| **asleep** | 0 | 24 | Hours |  | 15918 |
| **awake** | 0 | 24 | Hours |  | 3556 |
| **CardiacEffort** | 0 | 100 | beats / m | 10321 | 95170 |

***Table S14****: description and aggregations to compute daily measurements for each variable*

|  | **Description** | **Aggregation** |
| --- | --- | --- |
| **stepCount** | Number of daily steps | sum |
| **flightsClimbed** | Number of daily flights of stairs climbed | sum |
| **FlightsClimbedPaceMax** | Maximum pace at which stairs were climbed | max |
| **FlightsClimbedPaceMean** | Mean pace at which stairs were climbed | mean |
| **StepCountPaceMax** | Maximum gait speed | max |
| **StepCountPaceMean** | Mean gait speed | mean |
| **basalEnergyBurned** | Basal energy burned in a day | sum |
| **activeEnergyBurned** | Active energy burned in a day | sum |
| **appleStandTime** | Number of hours standing | sum |
| **walkingHeartRateAverage** | Heart rate when walking or running | mean |
| **heartRate** | Mean heart rate in a day | mean |
| **restingHeartRate** | Mean heart rate while at rest | mean |
| **heartRateVariabilitySDNN** | Variability in the gaps between heart beats | mean |
| **vo2Max** | Maximum volume of oxygen your body uses during exercise | mean |
| **Height** | Persons’s height | - |
| **BodyMass** | Person’'s weight | - |
| **distanceWalkingRunning** | Distance walked in a day | sum |
| **InBed** | Time in bed | sum |
| **asleep** | Time asleep | sum |
| **awake** | Time awake | sum |
| **CardiacEffort** | Amount of heart beats needed to walk 1 metre | mean |
| **BedBound** | An indication of whether a person spends most of their day in bed | sum |
| **Pace over threshold of maximum effort (70%)** | Amount of time in a day a person spends above 70% of their maximum capacity | mean |
